# Nucleoporin TPR promotes tRNA nuclear export and protein synthesis in lung cancer cells

**DOI:** 10.1371/journal.pgen.1009899

**Published:** 2021-11-18

**Authors:** Miao Chen, Qian Long, Melinda S. Borrie, Haohui Sun, Changlin Zhang, Han Yang, Dingbo Shi, Marc R. Gartenberg, Wuguo Deng

**Affiliations:** 1 State Key Laboratory of Oncology in South China and Collaborative Innovation Center for Cancer Medicine, Sun Yat-sen University Cancer Center, Guangzhou, Guangdong, China; 2 Department of Biochemistry and Molecular Biology, Robert Wood Johnson Medical School, Piscataway, New Jersey, United States of America; 3 Department of Obstetrics and Gynecology, The Seventh Affiliated Hospital of Sun Yat-sen University, Shenzhen, Guangdong, China; 4 The Cancer Institute of New Jersey, Rutgers University, New Brunswick, New Jersey, United States of America; University of California Davis, UNITED STATES

## Abstract

The robust proliferation of cancer cells requires vastly elevated levels of protein synthesis, which relies on a steady supply of aminoacylated tRNAs. Delivery of tRNAs to the cytoplasm is a highly regulated process, but the machinery for tRNA nuclear export is not fully elucidated. In this study, using a live cell imaging strategy that visualizes nascent transcripts from a specific tRNA gene in yeast, we identified the nuclear basket proteins Mlp1 and Mlp2, two homologs of the human TPR protein, as regulators of tRNA export. TPR expression is significantly increased in lung cancer tissues and correlated with poor prognosis. Consistently, knockdown of TPR inhibits tRNA nuclear export, protein synthesis and cell growth in lung cancer cell lines. We further show that NXF1, a well-known mRNA nuclear export factor, associates with tRNAs and mediates their transport through nuclear pores. Collectively, our findings uncover a conserved mechanism that regulates nuclear export of tRNAs, which is a limiting step in protein synthesis in eukaryotes.

## Author summary

Protein synthesis is a fundamental biological process for cells to grow and proliferate. Amino acids are the building blocks for proteins, and each is carried to ribosomes for protein synthesis by its own specific transport RNAs (tRNAs). An imbalanced or malfunctioning pool of cellular tRNAs may cause uncontrolled cell growth and proliferation, which are hallmarks of cancer. Nuclear pore complexes (NPCs) consist of multiple proteins termed nucleoporins that mediate transport of materials between the nucleus and the cytoplasm. Our previous study discovered that tRNA genes in yeast associate with NPCs to coordinate tRNA synthesis with nuclear export. Here, we develop a live cell imaging strategy to elucidate the machinery for tRNA nuclear export, and identify the nucleoporin TPR as a regulator of the process. We find that TPR is overexpressed in lung cancer tissues and correlated with poor prognosis, whereas down-regulation of TPR inhibits tRNA nuclear export, protein synthesis and cell growth. We further show that the nuclear export factor NXF1 associates with tRNAs and mediates their transport through NPCs. Our findings uncover a conserved mechanism by which NPCs control the quantity and quality of tRNAs before they reach the cytoplasm to execute their roles in protein synthesis.

## Introduction

Protein synthesis levels are tightly controlled to match the demands of cell growth. The demand for increased protein synthesis is greatest during the uncontrolled proliferation of cancer cells. Thus, understanding and harnessing the rate limiting steps in protein synthesis represents an important goal in cancer research. Studies have shown that the cellular tRNA pool plays an important role in the regulation of protein synthesis, and a role for tRNA abundance in cancer is emerging [1]. Not only is the size of the tRNA pool different in cancer cells but the content of specific tRNAs in that pool adjusts to selectively increase expression of genes required for cancer cell growth [2]. Differences in transcription of a subset of tRNA genes appear to drive the differences in tRNA content between cancerous and differentiated cells [3]. Importantly, a causal link between tRNA levels and cancer was suggested with the demonstration that artificial overexpression of the initiator methionine tRNA (tRNA_i_^Met^) promoted cell growth [4]. A more recent study found that tRNA^Glu(UUC)^ and tRNA^Arg(CCG)^ were upregulated in breast cancer cells, and enhanced the stability as well as translation of mRNA transcripts enriched for their codons, shifting the cellular proteomics to a pro-metastatic state [5].

Despite their small size, tRNA molecules follow a remarkably complex path to reach maturity before becoming aminoacylated. The detailed steps from synthesis by RNA polymerase III to maturity are perhaps best understood in the model organism budding yeast. In brief, the ends of the nascent transcripts are trimmed and a substantial number of bases are enzymatically-modified before the precursor tRNAs (pre-tRNAs) leave the nucleus. Following export, the precursors are spliced and bases are further modified before aminoacylation occurs [6]. Unlike yeast, the tRNA splicing machinery of mammalian cells is localized in the nuclei, and pre-tRNAs are spliced before exported to the cytoplasm [7].

The nuclear envelope is a barrier between the nucleus and cytoplasm that separates transcription from translation. Nuclear pore complexes (NPCs) are large protein structures that penetrate the nuclear envelope and permit the exchange of molecules between the nucleus and the cytoplasm. Each NPC is assembled from multiple copies of about 30 proteins termed nucleoporins that form distinct sub-structures, such as the core of the channel or the basket-shaped structure that protrudes into the nucleus [8]. In addition to nuclear-cytoplasmic transport, NPCs function in a variety of pivotal nuclear events, including chromosome segregation, chromatin organization and transcription regulation [9]. It is thus not surprising that many defects in NPCs are linked to tumorigenesis and development.

Translocated Promoter Region (TPR) protein is one of the first NPC components identified to be associated with cancer. In a chemically transformed human osteosarcoma-derived cell line, the N-terminal sequence of TPR was found to be fused to the kinase domain of the proto-oncogene MET, leading to the constitutive activation of MET [10]. Subsequent studies have reported fusions of TPR with kinases including TRK, FGFR1 and ALK in cancers [11–13]. However, TPR translocations are rare in human cancers [14]. Instead, emerging evidence suggests that the oncogenic role of TPR is related to its function in the nuclear export of macromolecules as a nuclear basket protein [15]. TPR has been found to interact with exportin 1 (XPO1, known as Crm1 in yeast) and nuclear export factor 1 (NXF1, also called TAP in human and Mex67 in yeast) [16,17]. Down-regulation of TPR was reported to disrupt the XPO1-dependent protein export [15,18], but enhance the NXF1-mediated export of mRNAs with retained introns [19]. Noteworthily, recent studies found no evidence for TPR to be essential to the nuclear retention of unspliced RNAs [20,21].

Studies in yeast have found several pathways for the nuclear export of nascent tRNAs, which are respectively mediated by Los1, Crm1 and Mex67-Mtr2 [22]. Among their human orthologs, exportin-t (XPOT/Los1) is a known nuclear export receptor for tRNA [23,24], and nuclear transport factor 2 like export factor 1 (NXT1/Mtr2) has been shown to stimulate nuclear export of tRNA *in vitro* [25]. However, it remains unclear whether XPO1 and NXF1 also function as tRNA nuclear exporters in vertebrates [22]. In this report, we first identify the yeast TPR homologs, Myosin-like proteins 1 and 2 (Mlp1 and Mlp2), to be regulators of tRNA nuclear export in yeast. We then show that human TPR plays the same role in lung cancer cells, which have elevated expression of TPR that are correlated with unfavorable prognosis in patients. We further demonstrate that NXF1, the interaction partner of TPR, mediates the transport of tRNAs through the NPCs. Finally, we show down-regulation of TPR causes nuclear accumulation of tRNAs, stalled protein synthesis, and inhibition of cell growth. Our results reveal a conserved mechanism by which eukaryotic cells regulate nuclear export of RNAs and protein synthesis.

## Results

### Mlp1/Mlp2 mediate the nuclear export of pre-tRNA in budding yeast

**Fig 1:**
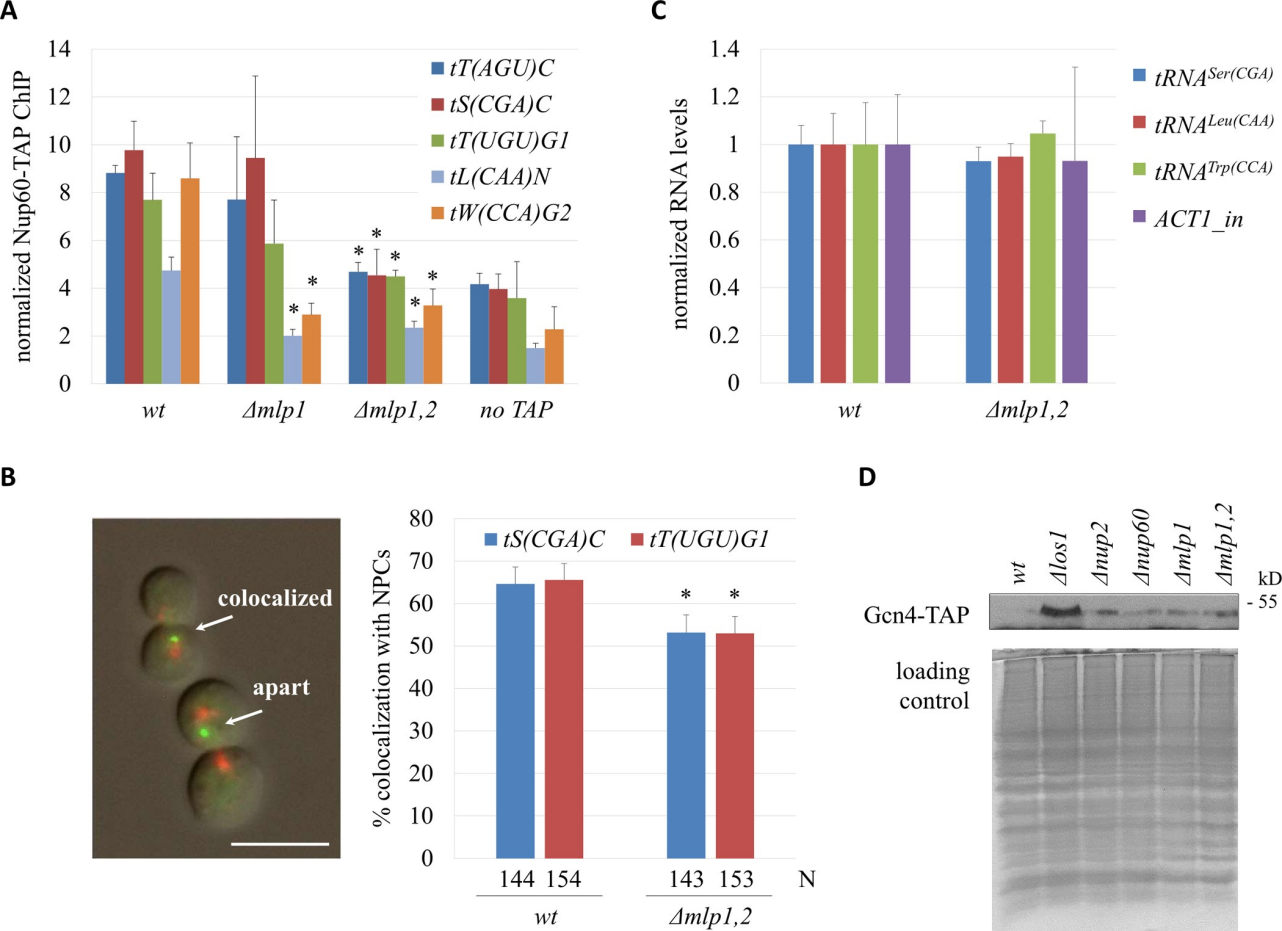
TPR orthologs in yeast contribute to tRNA export. (A) Binding of Nup60 at tRNA genes. ChIP-qPCR of Nup60-TAP was performed with strains MC177 (*wt*), MC273 (*Δmlp1*), MC276 (*Δmlp1*,*2*), and MC172 (*no TAP*) after M phase arrest by CDC20 depletion, when Nup60 was known to have increased association with tRNA genes in the *wt* strain. Data (mean ± SD, n = 3) were analyzed by Student’s *t*-test in paired comparison with the same tRNA gene in the *wt* strain; *P < 0.05. (B) tRNA gene-NPC association. Strains MC199 [*tS(CGA)C*::*256lac*^*op*^], MC200 [*tT(UGU)G1*::*256lac*^*op*^], MRG7425 [*tS(CGA)C*::*256lac*^*op*^ *Δmlp1*,*2*], and MRG7426 [*tT(UGU)G1*::*256lac*^*op*^ *Δmlp1*,*2*] were examined after M-phase arrest. Colocalization was defined as GFP and RFP foci were separated by no more than the width of the GFP spot. Scale bar = 10 μm. N denotes the number of cells analysed. Pairwise *χ*^2^-tests, *P < 0.05. (C) Nascent tRNA levels. RT-qPCR was done with strains MC177 (*wt*) and MC276 (*Δmlp1*,*2*) after arrest in M phase to measure the levels of three representative pre-tRNAs and nascent *ACT1* mRNA. Values were normalized to mature *ACT1* mRNA. (D) Western blot to detect the expression of Gcn4-TAP in asynchronously grown cultures of strains MC341 (*wt*), MC343 (*Δlos1*), MC349 (*Δnup2*), MC350 (*Δnup60*), MC344 (*Δmlp1*) and MC345 (*Δmlp1*,*2*). Coomassie blue staining was used to show even amount of protein loaded in gels.

Previously, we found that tRNA genes dock at NPCs when their expression peaks in M phase [26]. The behavior was reminiscent of several well-characterized RNA polymerase II-transcribed genes [27], and suggested that docking at NPCs coordinated tRNA production with nuclear export. Association of tRNA genes at NPCs required several factors, including nucleoporin Nup60 and nascent tRNA exportin Los1. Nup60 binds stably to the nuclear basket and anchors yeast myosin-like protein 1 (Mlp1) to the nucleoplasmic side of the NPC [28,29]. Mlp1 and its paralog Mlp2 are orthologs of the TPR protein in higher eukaryotes. The early finding that Mlp proteins associated with a subset of active RNA polymerase II-transcribed genes placed them at the nexus of transcription and NPC contact [30]. Thus, we investigated whether the Mlp proteins facilitated the association of tRNA genes with NPCs. ChIP-qPCR assays were performed to measure the association TAP-tagged Nup60 with tRNA genes in cells arrested in M phase by Cdc20 depletion. Values were normalized to the *GIT1* promoter that makes little contact with NPCs [26]. In wildtype (*wt*) cells, Nup60 was enriched at all five tRNA genes tested (Fig 1A). In the absence of Mlp1 (*Δmlp1*), contact was abolished for some tRNA genes (*tL(CAA)N* and *tW(CCA)G2*) but not for others. However, when both Mlp1 and Mlp2 were deleted, NPC contact with all of the tested tRNA genes was reduced to background levels. Thus, yeast TPR proteins are required for tRNA genes to contact fixed features of NPCs.

Fluorescence microscopy was used to investigate whether Mlp1/2 facilitate docking of tRNA genes at NPCs. To this end, we used strains that 1) target GFP-lacI near select tRNA genes, either *tS(CGA)C* or *tT(UGU)*G1, and 2) express RFP-tagged nucleoporin Nic96. The N-terminus of Nup133 was also deleted to cause NPCs to aggregate in one or more clusters on the nuclear membrane. Colocalization of the tRNA genes (green spots of fluorescence) and NPC clusters (red spots of fluorescence) was measured in cells arrested in M phase by Cdc20 depletion. Fig 1B shows that the two tRNA genes colocalize with NPCs but that colocalization drops significantly upon loss of Mlp1/2. The effect is not due to loss of tRNA gene expression. RT-qPCR analysis shows that nascent tRNA levels do not change when Mlp1/2 are deleted (Fig 1C). Collectively, these data demonstrate that yeast TPR proteins act redundantly in anchoring active tRNA genes to NPCs.

Our previous study suggested that docking of tRNA genes at NPCs increases the cytoplasmic pool of tRNA [26]. To better measure the impact of NPC-tRNA gene contact on pre-tRNA export from the nucleus, the intracellular distribution of nascent tRNA must be determined directly. To this end, we developed a new method to measure the distribution of unspliced tRNAs in living cells. The approach relies on an earlier strategy to visualize mRNAs that utilizes binding of the MS2 coat protein of bacteriophage to its RNA binding site [31]. In our system, mutants that block the export of a tRNA bearing MS2 binding sites should drive sequestration of MS2-GFP in the nucleus.

**Fig 2:**
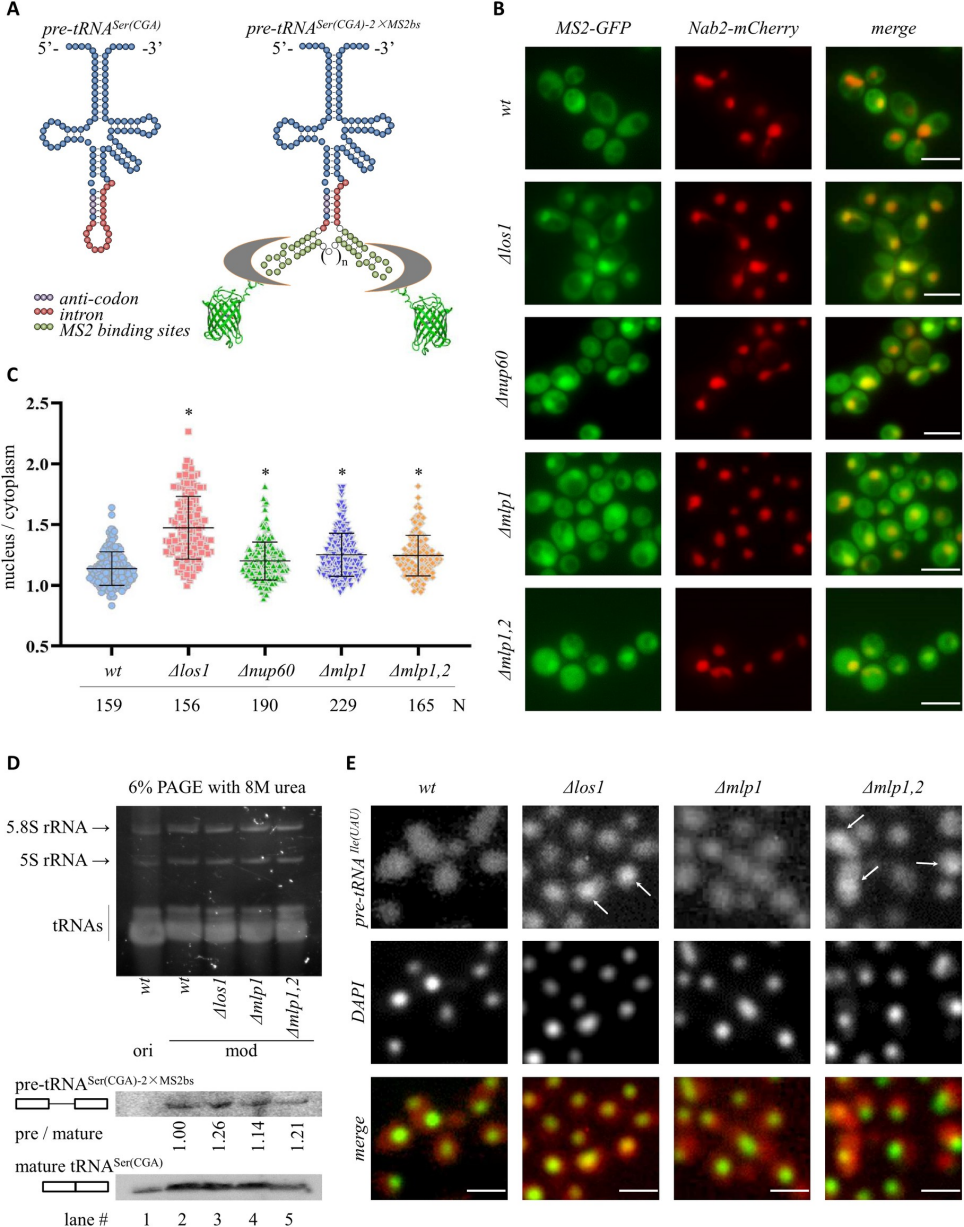
Mlp1/2 are required for tRNA export. (A) Schematics of unprocessed pre-tRNA^Ser(CGA)^ with and without synthetic MS2 sites. Color code of nucleotides is shown. The MS2 sites are bound by an MS2-GFP chimera that is expressed in yeast. The intron containing MS2 sites is spliced from the tRNA when it reaches the mitochondrial surface in the cytoplasm. (B) Representative images of cells expressing pre-tRNA^Ser(CGA)^ with MS2 sites, MS2-GFP, as well as Nab2-mCherry to image the nucleus. Asynchronously grown cultures of strains MRG5788 (*wt*), MRG5789 (*Δlos1*), MRG5787 (*Δnup60*), MRG5816 (*Δmlp1*), and MRG5817 (*Δmlp1*,*2*) were used. Scale bar = 10 μm. (C) Quantification was done by calculating the ratio of nuclear / cytoplasmic GFP signal intensity and shown in the column scatter graph. N denotes the number of cells examined, and *P < 0.05 by Student’s *t*-test when compared with the *wt* strain. (D) Nucleic acid staining (top) and Northern analysis (bottom) of RNAs isolated from yeast cells carrying the original wildtype *tS(CGA)C* [*ori*, strain MRG7400 (*wt*)] or the modified *tS(CGA)C*-*2×MS2bs* [*mod*, strains MRG5788 (*wt*), MRG5789 (*Δlos1*), MRG5816 (*Δmlp1*) and MRG5817 (*Δmlp1*,*2*)] gene. A single 3’-DIG labeled LNA-modified oligonucleotide probe was used to detect both pre-tRNA^Ser(CGA)-2×MS2bs^ (pre, 5-minute exposure) and mature tRNA^Ser(CGA)^ (mature, 20-second exposure). The ratio of pre / mature is indicated below each lane. A single 2-minute exposure of the whole blot is shown in S1B Fig. (E) FISH analysis for pre-tRNA^Ile(UAU)^ (red in the merged image) in strains BY4741 (*wt*), MC343 (*Δlos1*), MC344 (*Δmlp1*) and MC345 (*Δmlp1*,*2*). DAPI staining (green in the merged image) of DNA shows the nuclei. Scale bar = 10 μm.

For the assay, we replaced part of the 19 bp intron of the *tS(CGA)C* gene encoding tRNA^Ser(CGA)^ with a pair of MS2 binding sites (referred to as *tS(CGA)C-2×MS2bs*) in a yeast strain that expresses the MS2-GFP (Fig 2A). As this gene is the only source of tRNA^Ser(CGA)^ in yeast, the viability of the resulting strain indicates that the modified gene is still functional (S1A Fig, left). Indeed, growth rates of yeast are largely unchanged by the tRNA gene modification (S1A Fig, right).

In our live cell imaging system, MS2-GFP fluorescence was found throughout wildtype cells (Fig 2B). A subtle enrichment was found in the nucleus, which was demarcated by a nuclear protein tagged with mCherry (the NLS of Nab2 fused to mCherry). By contrast, strong nuclear fluorescence was found in mutants lacking the tRNA export factor Los1 (Fig 2B), consistent with a tRNA export defect. The experiment was repeated in mutants that abolish contact between tRNA genes and NPCs. In strains lacking Nup60, Mlp1 or Mlp1/2, MS2-GFP was also enriched in the nucleus (Fig 2B and 2C). The enrichments depended on MS2 binding sites as MS2-GFP was evenly distributed between the nucleus and the cytoplasm of *wildtype*, *Δlos1* and *Δmlp1*,*2* cells that lack the MS2bs-modified tRNA (S2 Fig).

Northern blotting was performed to evaluate the impact of the modified intron on pre-tRNA processing and mature tRNA production. This analysis was necessary to determine whether there were any artefacts that would obscure the interpretation of the live cell imaging assay. Blotting with a locked nucleic acid (LNA)-modified oligonucleotide probe to the mature tRNA^Ser(CGA)^ showed that the modified *tS(CGA)C-2×MS2bs* gene produces roughly wild-type levels of tRNA^Ser(CGA)^, thereby confirming that the modified gene is a suitable substitute for the original (Compare lanes 1 and 2 of Fig 2D). A longer exposure of the same blot showed that the modified gene also yields detectable amounts of pre-tRNA whereas the unmodified gene does not (See S1B Fig, left panel). The unmodified pre-tRNA could only be detected with an even longer exposure and higher levels of RNA extracts (See S1B Fig, right panel). These data indicate that incorporation of MS2 sites produces changes in the steady-state level of the low abundance pre-tRNA. We took advantage of the pre-tRNA^Ser(CGA)-2×MS2bs^ signal to evaluate mutants. Recall that tRNA splicing occurs in the cytoplasm. Mutants that block nuclear export of pre-tRNAs will lead to enrichment of unspliced precursors. Fig 2D shows that the ratio of pre-tRNA^Ser(CGA)-2×MS2bs^ / tRNA^Ser(CGA)^ increased in cells lacking Los1 or the Mlp proteins. A second northern blot with a probe to detect just the 2×MS2 binding sites reinforced this conclusion. This probe hybridized to both the pre-tRNA^Ser(CGA)-2×MS2bs^ and the spliced intron, showing that the pre-tRNA^Ser(CGA)-2×MS2bs^ / 2×MS2bs ratio also increased in *Δlos1* and *Δmlp1*,*2* cells (S1C Fig). Taken together, these results indicate that Los1 and the Mlps participate in tRNA export, thus validating the results of our live cell MS2-GFP assay.

We compared our live MS2-GFP imaging approach to tRNA fluorescence *in situ* hybridization (tRNA-FISH), an established method of detecting the intracellular location of tRNAs [32]. A probe to pre-tRNA^Ile(UAU)^ was used, thereby allowing the evaluation of an entirely unrelated and unmodified tRNA. The images in Fig 2E show that the pre-tRNA accumulates densely in the nuclei of cells lacking Los1 and both Mlp proteins, albeit less so in cells lacking only Mlp1. Thus, the traditional and more laborious FISH method yields parallel results to our live assay based on nuclear sequestration of MS2-GFP. Both show a role for Los1 and the Mlp proteins in tRNA export. The data above collectively indicate that anchoring of tRNA genes at NPCs promotes efficient export of the unspliced tRNA out of the nucleus, and that unspliced tRNAs accumulate in the nuclei of cells lacking the yeast TPR proteins.

Gcn4 is a transcription activator that is translated in response to starvation or stress to slow down general protein synthesis [33]. Noteworthily, accumulation of pre-tRNA in the nucleus due to deletion of Los1 also activates Gcn4 [34]. Accordingly, we examined whether other mutants defective in pre-tRNA nuclear export also induced Gcn4 expression. Fig 1D shows that the protein level of Gcn4 was increased in strains lacking Los1, Nup2, Nup60, Mlp1 or Mlp1/2, indicating that cellular protein synthesis was repressed when tRNA nuclear export was impaired. Collectively, our data suggest that yeast TPR proteins coordinate tRNA gene transcription with nuclear export to regulate protein synthesis in budding yeast.

### The expression of TPR is elevated in lung cancer

**Fig 3:**
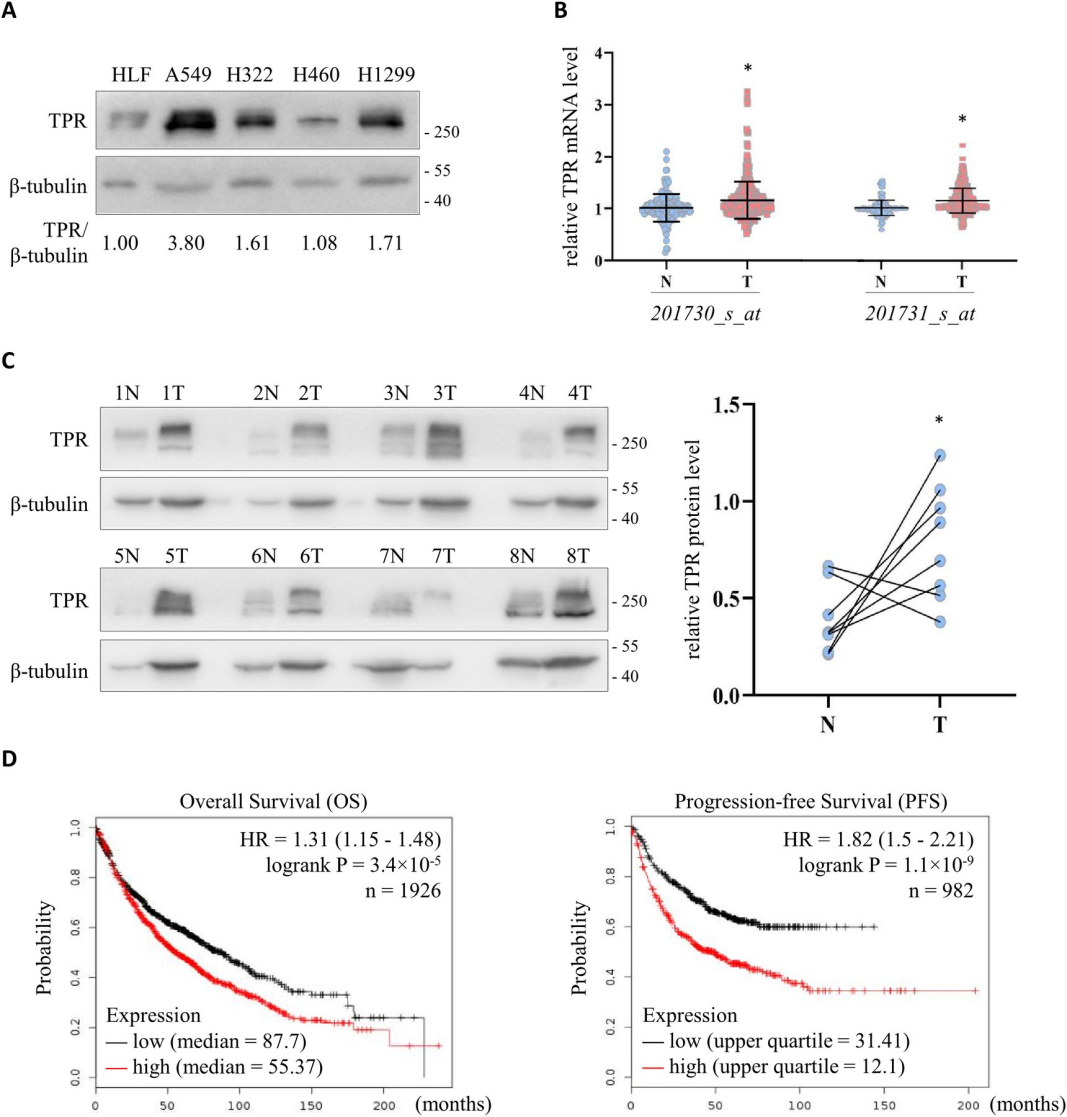
Elevated expression of TPR in lung cancer cell lines and tissues. (A) Western blot to detect the expression of TPR in lung cancer cell lines. HLF (human lung fibroblast) served as the normal control, while A549 (lung adenocarcinoma cell), H322 (bronchioalveolar carcinoma cell), H460 (large cell lung cancer cell) and H1299 (lung carcinoma cell) were representative lung cancer cells. β-tubulin was used as the loading control. (B) TPR mRNA level in human lung cancer (T, n = 356) and non-tumor (N, n = 161) tissues. Data were extracted from GSE7670, GSE10072, GSE19188 and GSE31210, normalized to the median value of the non-tumor tissues in each dataset, and compiled for analysis. *P < 0.05 by Student’s *t*-test. (C) Left: Western blot to detect the expression of TPR in paired tumor (T) and adjacent non-tumor (N) tissues from lung cancer patients. β-tubulin served as the loading control. Right: Quantification of TPR expression relative to β-tubulin in patient samples. *P < 0.05 by Student’s *t*-test for pairwise comparison. (D) The prognostic value of TPR mRNA level in lung cancer patients from KM plotter. Overall survival (OS, left) and progression-free survival (PFS, right) curves were plotted using 201731_s_at, which was selected as the optimal microarray probe to represent TPR by JetSet. The low and high TPR expression patient cohorts were compared by a Kaplan-Meier survival analysis, and the hazard ratio (HR) with 95% confidence intervals and logrank P value were calculated.

Our query of ONCOMINE (https://www.oncomine.org) [35] showed that human TPR mRNA levels are often elevated in cancers (S3A Fig). Therefore, we examined the mRNA level of TPR in the NCI-60 cancer cell lines from GSE5720 [36] and GSE5846 [37] with two independent datasets of genome-wide expression profiles using the Affymetrix HG-U133A array. The expression of TPR detected by two different probes (201730_s_at and 201731_s_at) was analyzed according to the tissue origins of the cells (Table C-D in S1 Text). Lung cancer cells consistently had high levels of TPR mRNA (S3C Fig). We also investigated the protein abundance of TPR using the NCI-60 proteome resource (http://wzw.tum.de/proteomics/nci60) [38]. We found that, like mRNA levels, the expression of TPR protein was elevated in lung cancer cells (Table E in S1 Text and S3B and S3C Fig). In addition, western blotting showed that TPR was elevated in A549, H322, H460 and H1299 lung cancer cells compared to normal human lung fibroblasts (HLF) (Fig 3A).

To further explore the clinical significance of TPR expression, we carried out a compiled analysis of TPR mRNA abundance in tumor and adjacent tissues from lung cancer patients using independent datasets GSE7670, GSE10072, GSE19188 and GSE31210 [39–41]. This comparison indicated that TPR mRNA was increased in tumor tissues (Fig 3B). In accordance with this result, western blotting of 8 pairs of tumor and adjacent tissues from lung cancer patients confirmed that the protein level of TPR was higher in tumor tissues (Fig 3C). To analyze the correlation between TPR mRNA level and different clinicopathological features, we queried three independent lung cancer patient datasets: GSE4573 [42], GSE8894 [43] and GSE12667 [44]. Pearson’s *χ*^2^ test indicated that smoking was associated with higher TPR expression (*P* = 0.0433), while the expression of TPR positively correlated with cancer grade (*P* = 0.0161). Moreover, tumors with larger volumes (>50 mm) and tumors with regional lymph node metastasis were shown to have higher TPR level (Table F in S1 Text). Finally, we explored the prognostic value of TPR mRNA expression in the Kaplan-Meier plotter database (http://www.kmplot.com/lung/) [45]. We found that lung cancer patients with high TPR mRNA had shorter overall survival and progression-free survival (Fig 3D). Altogether, the analyses above imply that the expression of TPR is increased in lung cancer cells and tissues, with higher levels corresponding to worse prognoses.

**Fig 4:**
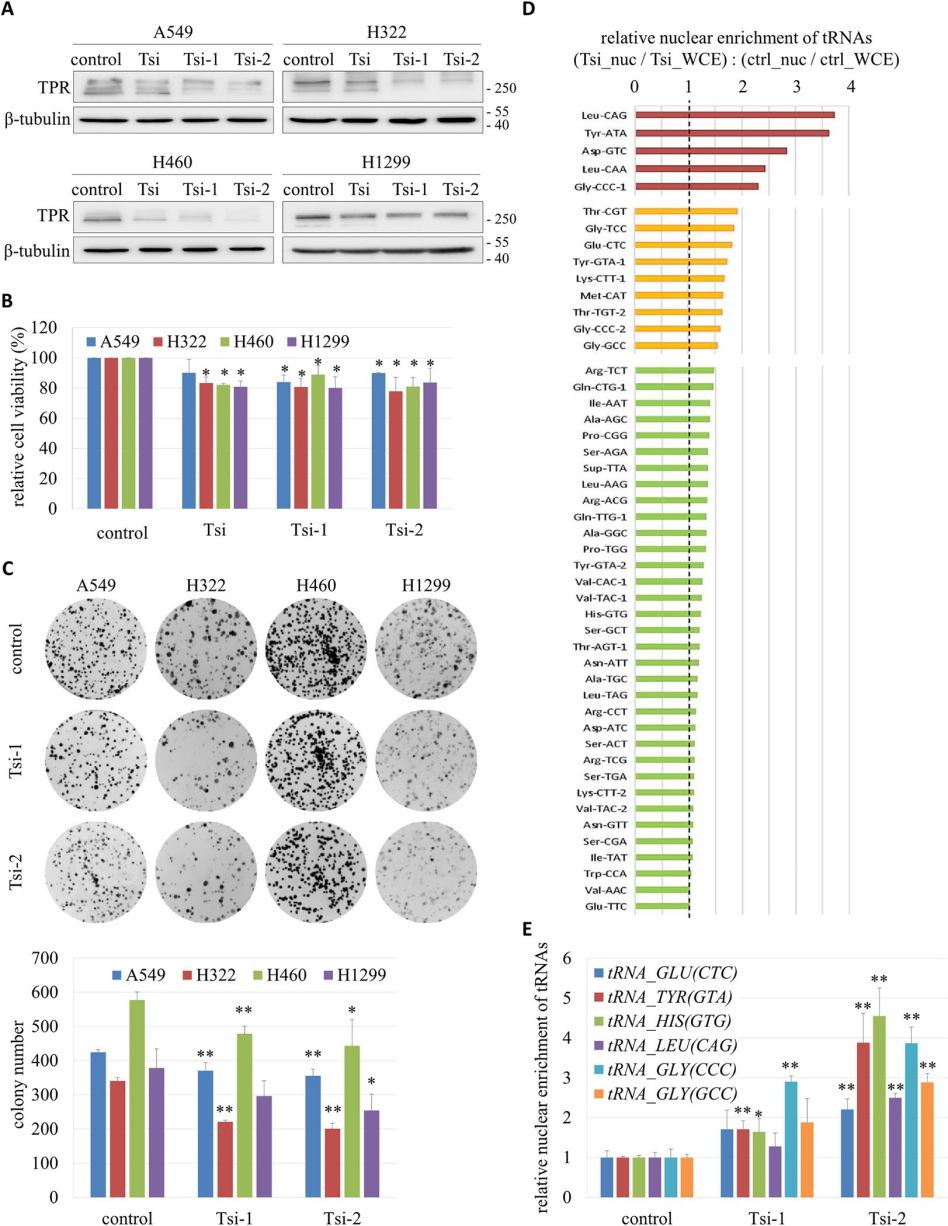
Growth inhibition and nuclear accumulation of tRNAs in lung cancer cells with TPR knockdown. (A) Western blot of TPR in A549, H322, H460 and H1299 cells transfected with TPR-specific siRNAs (Tsi, Tsi-1 and Tsi-2) or a control siRNA. (B) Cell viability measured by MTS assay. Data (mean ± SD, n = 3) were analyzed by paired Student’s *t*-test; *P < 0.05. (C) Cell proliferation capacity evaluated by colony formation assay. Representative images were shown on the top panel. Quantification was presented on the bottom panel. Data (mean ± SD, n = 3) were analyzed by paired Student’s *t*-test; *P < 0.05, **P < 0.01. (D) Nuclear enrichment of tRNAs in TPR knockdown cells relative to control cells. The nrStar Human tRNA PCR Array was used to measure the levels of nuclear-encoded tRNAs and U6 small nuclear RNA included in the array in the nuclear (ctrl_nuc) and whole cell extracts of A549 cells (ctrl_WCE), as well as A549 cells after TPR knockdown (Tsi_nuc and Tsi_WCE, respectively). The values of the tRNAs were normalized to that of U6, which was known to remain in the nucleus after transcription by RNA polymerase III. Nuclear enrichment of each tRNA in Tsi cells relative to control cells was reported as the ratio of (Tsi_nuc/Tsi_WCE): (ctrl_nuc/ctrl_WCE), as shown in the graph. (E) Direct measurement of nuclear tRNA enrichment by RT-qPCR. Nuclear and whole cell RNA extracts were prepared from A549 cells (control) and A549 cells with TPR knockdown (Tsi-1 and Tsi-2). RT-qPCR was performed for each extract to measure the level of tRNA^Glu(CTC)^, tRNA^Tyr(GTA)^, tRNA^His(GTG)^, tRNA^Leu(CAG)^, tRNA^Gly(CCC)^ and tRNA^Gly(GCC)^, using U6 as an internal control. Relative nuclear enrichment of each tRNA was determined by its nuclear / total expression normalized to the same ratio of the tRNA within the control. Data (mean ± SD, n = 3) were analyzed by paired Student’s *t*-test; *P < 0.05, **P < 0.01.

To investigate whether TPR promotes the growth of lung cancer cells, we transiently knocked down the protein using three different siRNAs in A549, H322, H460 and H1299 cells (Figs 4A and S4A). An MTS assay to assess cell metabolic activity indicated that the viability of cells was reduced when TPR was down-regulated (Fig 4B). Also, a colony formation experiment demonstrated that knockdown of TPR strongly inhibited the proliferation of three of the four lung cancer cell lines (Fig 4C). However, our flow cytometry analysis showed that down-regulation of TPR did not cause dramatic defects in cell cycle progression or chromosome segregation during mitosis (S4B Fig).

### TPR promotes tRNA nuclear export in lung cancer cells

Considering that the yeast orthologs of TPR coordinated the coupled transcription and nuclear export of pre-tRNA for protein synthesis in yeast (Figs 1 and 2), we hypothesized that elevated expression of TPR facilitated the nuclear export of tRNAs in lung cancer cells. To test this hypothesis, we investigated whether TPR knockdown increased the levels of tRNAs in the nucleus. To this end, we measured the nuclear enrichment of 66 human nuclear encoded tRNAs in A549 cells using the nrStar Human tRNA PCR Array, as described in the Materials and Methods. The levels in nuclear extracts were normalized to those in whole cell extracts. Fig 4D shows that 48 (over 70%) of the tested tRNAs were more enriched in the nucleus when the expression of TPR was knocked down with the Tsi siRNA, and the top 10 tRNAs enriched in the nucleus included tRNAs carrying hydrophobic (Leu), polar (Tyr, Thr), charged (Asp, Glu, Lys) and small (Gly) amino acids. The relative levels of the tRNAs in TPR knockdown and control cells had a strong positive linear relationship (S5A Fig), suggesting that TPR did not affect the transcription of certain subsets of tRNAs.

RT-qPCR was performed to validate that loss of TPR inhibited the nuclear export of tRNAs. Fig 4E shows that the nuclear levels of tRNA^Glu(CTC)^, tRNA^Tyr(GTA)^, tRNA^His(GTG)^, tRNA^Leu(CAG)^, tRNA^Gly(CCC)^ and tRNA^Gly(GCC)^ increased in A549 cells upon knockdown of TPR. Similar results were found for H322 and H1299 cells (S5B Fig). Noteworthily, knockdown of TPR caused no change in the total abundance of tRNAs (S6A Fig), nor did it affect the transcription of tRNAs, which was reflected by the levels of nascent pre-tRNAs (S6B Fig). Our experiments indicate that TPR normally promotes tRNA nuclear export in lung cancer cells.

### NXF1 mediates TPR-regulated tRNA nuclear export

**Fig 5:**
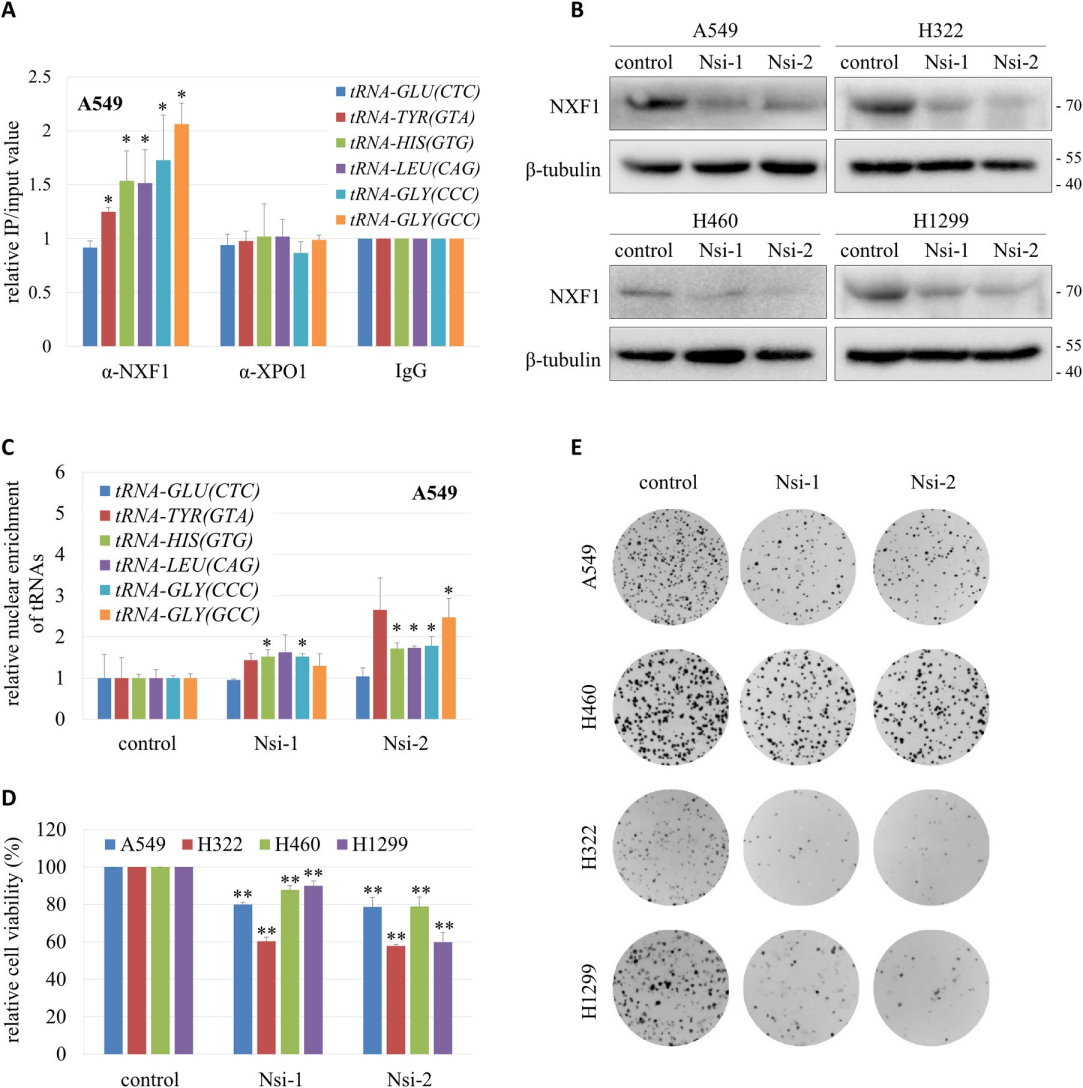
A role for NXF1 in tRNA nuclear export. (A) Interaction between NXF1 and tRNAs. RIP assay was performed with α-NXF1 or α-XPO1 on A549 cell lysates. RIP with IgG served as control. RT-qPCR was performed to measure the amount of tRNA^Glu(CTC)^, tRNA^Tyr(GTA)^, tRNA^His(GTG)^, tRNA^Leu(CAG)^, tRNA^Gly(CCC)^ and tRNA^Gly(GCC)^ in the precipitates. Data (mean ± SD, n = 3) were analyzed by Student’s *t*-test in paired comparison; *P < 0.05. (B) Western blot of NXF1 in A549, H322, H460 and H1299 cells transfected with NXF1-specific siRNAs (Nsi-1 and Nsi-2) or a control siRNA. (C) Nuclear enrichment of tRNAs in A549 cells (control) and A549 cells with NXF1 knockdown (Nsi-1 and Nsi-2). Relative nuclear enrichment of each tRNA tested was determined by its nuclear / total expression normalized to the same ratio of the tRNA within the control, shown as mean ± SD (n = 2), and analyzed by Student’s *t*-test; *P < 0.05. (D) Cell viability measured by MTS assay. Data (mean ± SD, n = 3) were analyzed by paired Student’s *t*-test; **P < 0.01. (E) Cell proliferation capacity evaluated by colony formation assay.

Yeast Mex67 and Crm1 (NXF1 and XPO1 in human) are involved in the nuclear export of tRNAs [46]. To test whether NXF1 and XPO1 are exporters of tRNAs in lung cancer cells, we performed RNA immunoprecipitation (RIP) with NXF1 and XPO1 antibodies respectively. Fig 5A shows that most of the tRNAs tested co-immunoprecipitated with NXF1 but not XPO1. To further verify that NXF1 is involved in the nuclear export of tRNAs, NXF1 was knocked down by specific siRNAs in lung cancer cell lines (Fig 5B). RT-qPCR showed that decreased expression of NXF1 caused nuclear accumulation of the various tRNA species examined (Figs 5C and S7A).

The viability and proliferation capacity of lung cancer cells were dramatically reduced when NXF1 was knocked down (Figs 5D–5E and S7B), similar to the effects of TPR knockdown (Fig 4). However, unlike TPR, the mRNA and protein level of NXF1 was not increased in tumor tissues from lung cancer patients (S8A and S8B Fig), and knockdown of TPR did not down-regulate the expression of NXF1 (S8C Fig). These data indicate that TPR does not regulate tRNA nuclear export through modulating NXF1 expression. Instead, TPR may facilitate the association of NXF1 with the NPC to promote NXF1-mediated tRNA nuclear export.

### TPR promotes RNA export and protein synthesis

**Fig 6:**
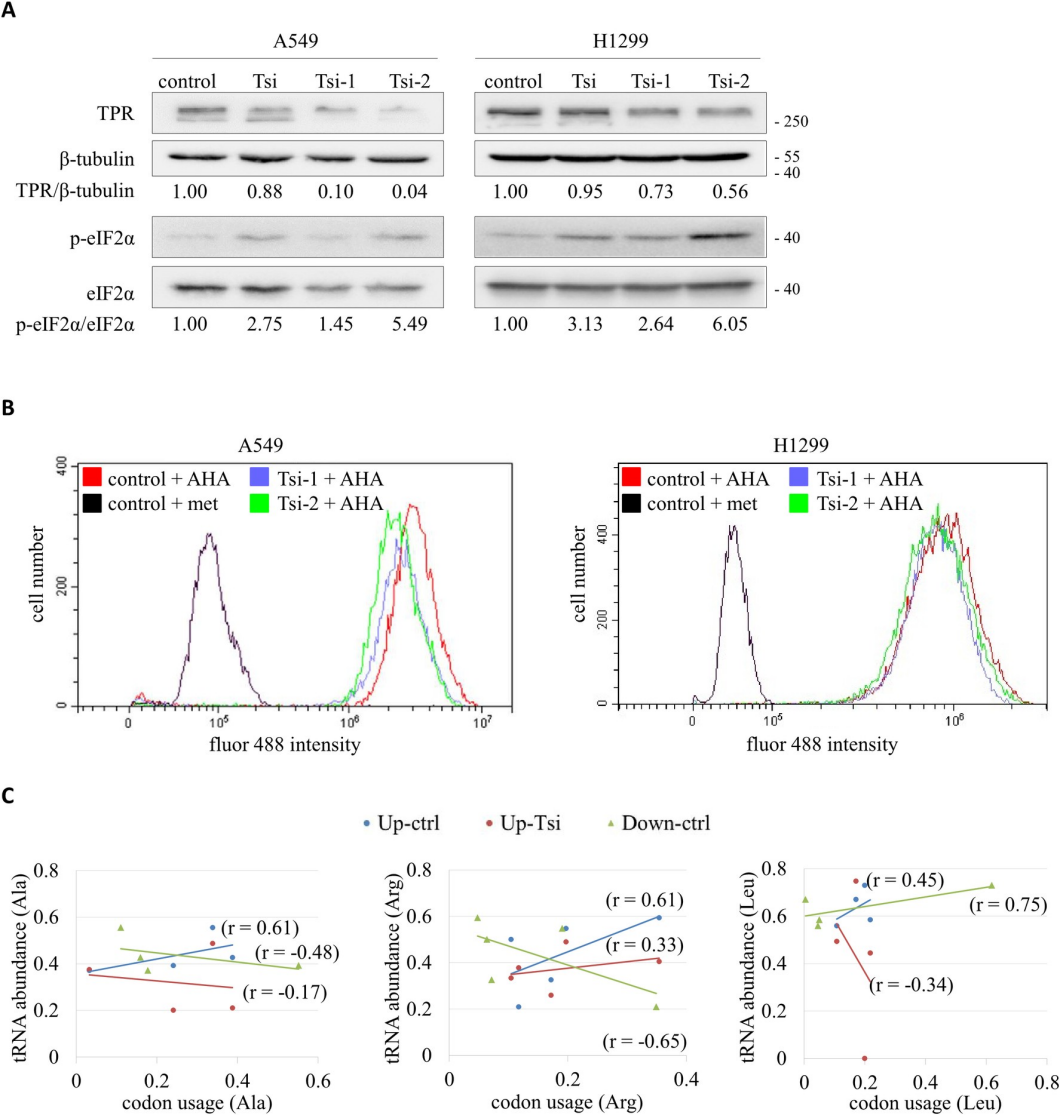
Suppression of protein synthesis by TPR knockdown. (A) Western blot to detect the phosphorylation of eIF2α. Protein extracts were prepared from A549 and H1299 cells without (control) or with TPR knockdown (Tsi, Tsi-1 and Tsi-2). Specific antibodies were used to detect the cellular levels of TPR, β-tubulin, phosphorylated eIF2α (p-eIF2α) and total eIF2α. Numbers indicate the ratios of TPR / β-tubulin (top) and p-eIF2α / eIF2α (bottom). (B) FUNCAT assay followed by flow cytometry analysis of the fluor 488 signal intensity. A549 and H1299 cells without (control) or with TPR knockdown (Tsi-1 and Tsi-2) were incubated with 4 mM azidohomoalanine (AHA) or methionine (met, as negative control) for 2 hours, and tagged with fluor 488-alkyne for detection. At least 10,000 cells were analyzed for each condition. (C) Correlation of cytoplasmic abundance of tRNA^Ala^, tRNA^Arg^ and tRNA^Leu^ isoacceptors in control (ctrl) and TPR knockdown (Tsi) A549 cells to codon usage of the top 10 up-regulated (Up) and the 10 most down-regulated (Down) genes in lung cancer tissues with high TPR expression. Cytoplasmic abundance of tRNA was calculated as tRNA_cyt_ = tRNA_tot_ - tRNA_nuc_, using the data from the tRNA PCR array.

The eukaryotic initiation factor 2 (eIF2) is an important effector of cellular stress responses and a key regulator of global mRNA translation [47]. Phosphorylation of the α-subunit of eIF2 (eIF2α) on serine 51 hampers the turnover of the eIF2-bound GDP to GTP, thus impeding the formation of the eIF2-GTP-tRNA^iMet^ ternary complex and the initiation of protein synthesis [48]. Our immunoblots in Fig 6A show that phosphorylated eIF2α (p-eIF2α) was elevated relative to eIF2α in A549 and H1299 cells following transient knockdown of TPR. The same results were also observed in H322 and H460 cells (S9A Fig). As a complementary strategy, we performed a fluorescent noncanonical amino acid tagging (FUNCAT) assay to detect newly synthesized proteins by combination of azidohomoalanine (AHA) incorporation and click chemistry labeling. In TPR-knocked down A549 and H1299 cells, the amount of nascently generated proteins was diminished (Fig 6B). These results support the notion that TPR promotes protein synthesis in lung cancer cells.

Given that TPR also functions in the nuclear export of 5.8S rRNA (S10 Fig), mRNAs [49], and lncRNA [20], we hypothesized that TPR might orchestrate the nuclear export of various RNAs for translation in cells. To test this idea, we analyzed the correlation between the abundance of cytoplasmic tRNAs to the codon usage of the top 10 genes (Spearman’s correlation > 0.63, *P* < 0.001), the mRNA expression of which is positively correlated to that of TPR based on the analysis of 510 lung adenocarcinoma patient samples from the TCGA database. The coding sequences for these gene transcripts were compiled and analyzed for codon content with the Sequence Manipulation Suite (http://bioinformatics.org/sms). Positive correlations were found between the levels of all tRNAs tested and codon usage for the selected genes (S9B Fig). Data for five representative amino acids with over three isoacceptors (tRNA^Ala^, tRNA^Arg^, tRNA^Leu^, tRNA^Pro^ and tRNA^Ser^) are shown in Figs 6C and S9C. Following TPR knockdown, the positive correlations were lost for tRNA^Ala^, tRNA^Leu^ and tRNA^Ser^, and reduced for tRNA^Arg^ and tRNA^Pro^. Also included were analyses for the top 10 genes (Spearman’s correlation < -0.52, *P* < 0.001) with mRNA expression negatively correlated to that of TPR, which showed inverse (tRNA^Ala^, tRNA^Arg^, tRNA^Pro^ and tRNA^Ser^) or weaker (tRNA^Leu^) tRNA abundance-codon usage correlations in control cells. These data suggest that TPR enhances protein synthesis by coordinating the nuclear export of varied RNAs in lung cancer cells.

## Discussion

The major findings of our current study are that (i) the yeast homologs of TPR, Mlp1 and Mlp2, are required for tRNA gene docking at NPCs and nuclear export of nascent tRNA transcripts; (ii) the tRNA export role for TPR is conserved in human lung cancer cells, and fulfilled through association with NXF1, an RNA binding factor that mediates export of both tRNA and mRNA through NPCs; (iii) TPR coordinates the nuclear export of various RNAs including mRNAs, tRNAs and rRNAs, and promotes protein synthesis in eukaryotes.

### MS2-GFP system to study the nuclear export of tRNAs in living cells

In order to detect the distribution of unspliced tRNAs in living cells, we have established a live cell imaging system that utilizes binding of the GFP-tagged MS2 coat protein to its RNA binding site. The system is easy to use and accurately recapitulates findings derived from the traditional and more laborious tRNA FISH approach. Using this system, we have identified mutants that hamper the export of a tRNA bearing MS2 binding sites [pre-tRNA^Ser(CGA)-2×MS2bs^] and thus sequester MS2-GFP in the nucleus (Fig 2).

It has been noted that the MS2-GFP system in mRNA studies caused artefactual accumulation of mRNA decay products [50,51]. In our application, however, we found that the mature tRNA is by far the dominant species (Figs 2D and S1B). Indeed, no other partial decayed tRNA fragments are observed. Moreover, we never see punctate patterns of fluorescence that are characteristic of the mRNA decay artefacts [51]. Thus, we believe our use of two binding sites in tRNA is free of the problems previously associated with the dozens of MS2 binding sites used to visualize mRNA.

Could the spliced intron be a problem? This seems unlikely. In our system, the MS2 binding sites replace all but 10 of the *tS(CGA)C* intronic nucleotides. Once spliced from the tRNA in the cytoplasm [52,53], the small fragment lacks features that would guide it (or the MS2-GFP bound counterpart) preferentially back to the nucleus. Proteins that transport and splice tRNAs with introns almost entirely recognize conserved features of the tRNA, not the highly varied intronic sequences [54,55]. The relatively uniform nuclear and cytoplasmic fluorescence in wildtype cells provides a base line from which to measure perturbations. Thus, nuclear enrichment of MS2-GFP in the mutants described here (Los1, Nup60 and Mlp1 and Mlp2) indicates that nuclear export of intronic pre-tRNAs has been compromised.

### TPR facilitates tRNA nuclear export

The 270 kD TPR protein is anchored to the nuclear basket on the nucleoplasmic side of the NPC by its NH_2_-terminal coiled coil domain. The acidic terminus of the protein is free to interact with transport factors and mediate the trafficking of macromolecules between the nucleus and cytoplasm [56]. The emerging picture from studies in a variety of organisms is that TPR plays a critical role in RNA quality control. In yeast, for example, the TPR ortholog Mlp1 retains faulty pre-mRNAs [28]. In mammalian cells, ectopic expression of TPR causes nuclear accumulation of poly(A)^+^ RNAs [56], and knockdown of TPR increases the nuclear export of mRNAs with introns [19]. However, it has been called into question recently that TPR is required for the nuclear retention of unspliced transcripts [21]. Lee *at al* showed that TPR is required for the nuclear export of both mRNAs and lncRNAs transcribed from intronless or intron-poor genes [20]. Similarly, here we find that TPR is required for the nuclear export of tRNAs, which either contain only a single intron or no intron at all (Fig 4). These studies indicate that TPR functions as a common docking site at the NPC to regulate the nuclear export of a wide variety of RNA species through its interaction with various export receptors and RNA binding proteins, such as NXF1 and components of the transcription and export complex 2 (TREX-2) complex [17,21,49].

Although TPR knockdown caused nuclear retention of most tRNAs (Fig 4D), we observed no significant change in their total amounts or the expression levels of the pre-tRNAs we tested (S6 Fig). Consistently, our double deletion of the TPR orthologs Mlp1 and Mlp2 in yeast had only a negligible effect on the level of the nascent tRNA transcripts examined (Fig 1C). Accordingly, TPR is unlikely to regulate the transcription of tRNAs directly, and the nuclear accumulation of tRNAs in TPR-depleted cells does not arise from elevated tRNA transcription in the nucleus. Instead, TPR probably serves as a major gateway for transport of tRNAs and other RNAs, which restricts unprocessed or aberrant RNAs to the nucleus for further processing or degradation, but promotes the nuclear export of properly processed RNAs, ensuring that only mature transcripts access the translation machinery in cytoplasm. This can be achieved through the interaction between TPR and a multitude of RNA adaptor proteins. For instance, TPR associates with the serine and arginine rich splicing factor SRSF5 [57], which retains its bound intron-containing mRNAs and noncoding RNAs to the nucleus when its arginine residues are hypomethylated, but recruits NXF1 to mRNAs to facilitate their export when hypermethylated [58].

### NXF1 mediates the nuclear export of tRNAs

Mex67 (ortholog of NXF1) was first shown to function in the nuclear export of a subset of tRNAs in yeast [22]. Subsequently, Mex67 in trypanosomatids was reported to selectively export tRNAs that are typically modified with queuosine (nucleoside Q), a guanosine derivative found only in tRNA^Asp^, tRNA^Asn^, tRNA^His^ and tRNA^Tyr^ [59]. Here, we demonstrated that NXF1 binds tRNAs in human lung cancer cells and mediates tRNA nuclear export (Figs 5 and S7). We noticed that among the six tRNA species tested, tRNA^Glu(CTC)^ was not bound by NXF1, as shown in the RIP experiment (Fig 5A). Consistently, knockdown of NXF1 caused no nuclear accumulation of tRNA^Glu(CTC)^ (Fig 5C). These findings suggest that NXF1 displays tRNA substrate preferences, whereas TPR serves as a general anchor for nuclear export receptors carrying different subsets of tRNAs.

Currently, it remains elusive how NXF1 binds tRNA species selectively. The protein contains two essential RNA-binding domains (RBDs) [60,61]. One RBD binds mRNAs with no obvious sequence specificity, yet adaptor proteins can increase both affinity and specificity [17]. The second RBD binds to the stem-loop structures in the constitutive transport elements of retroviral RNAs to mediate the nuclear export of unspliced viral RNAs [62]. One possibility is that this RBD targets similar stem-loop structures in tRNAs [22,63]. Another possibility is that NXF1 binds to tRNAs with unique post-transcriptional modifications, which can determine tRNA identity, influence tRNA folding and stability, and affect translation [64]. A recent study in mRNAs and lncRNAs discovered that NXF1 preferentially bound 5’-biased, G/C-rich and m^6^A-modified regions of transcripts with one or two exons [21]. In addition, the association between NXF1 and certain tRNAs might be mediated or stabilized by specific adaptor proteins. These scenarios are not mutually exclusive, and future investigations are needed to dissect the detailed mechanism by which NXF1 recognizes and exports tRNAs from nucleus to cytoplasm.

### Nuclear export of tRNAs affects protein synthesis

tRNAs play a central role in translation as each tRNA provides a direct and specific link between a triplet of nucleotides in mRNA and the corresponding amino acid. Recent findings indicate that tRNAs are not only adaptors in translation, but important molecules influencing protein expression in cell cycle progression [65], cell proliferation and differentiation [3], stress response [66], cancer and other diseases [67,68], as well as drug resistance formation [69]. At the same time, many pathways have been uncovered that control tRNA transcription, nascent tRNA processing, surveillance and turnover of mature and incompletely processes species [64]. It is known that the abundance of tRNAs can affect translation efficiency independently of factors such as mRNA abundance [66]. Overexpression of tRNAs in cancer can accelerate the rate of protein synthesis [2], whereas an imbalance between mRNA codon usage and the relative abundance of cognate tRNAs can impact the polypeptide elongation rate and induce pauses in translation [70]. In line with this, our analysis showed that abundance of cytoplasmic tRNAs was positively correlated with the codon usage in mRNA transcripts enriched in lung cancer tissues with high expression of TPR, but such a correlation was impaired when TPR was knocked down and the nuclear export of tRNAs was blocked (Figs 6C and S9C).

TPR and NXF1 have known roles in the nuclear export of mRNAs and lncRNAs [17,20,21,49]. Our finding of their functions in tRNA trafficking in lung cancer cells suggests that TPR and NXF1 orchestrate the nuclear export of different forms of RNAs to promote protein synthesis. Such multitasking is likely to streamline the genomes of cells, and serve as a regulatory strategy to ensure translation efficiency and fidelity. Moreover, a recent study in colorectal cancer cells reported that rapid depletion of TPR by the auxin-induced degron (AID) system not only disrupted association of the TREX-2 complex subunits to NPCs, but also resulted in pronounced changes in RNA transcription and export [49]. We used immunoblot to examine whether knockdown of TPR alters the expression of proteins involved in NPC functions, and found that XPO1 level was decreased when TPR was knocked down in A549 lung cancer cells (S11 Fig). Although our RIP experiment showed no association of XPO1 with tRNAs (Fig 5A), XPO1 is well known for its role in the nuclear export of ribosomal subunits [71,72]. Therefore, TPR can also impact tRNA biology and protein translation by indirect mechanisms, such as regulating the expression and localization of proteins related to NPC functions.

Notably, our analysis of lung cancer patient samples showed significantly higher expression of TPR in tumor than in adjacent tissues (Fig 3B and 3C), whereas NXF1 remained unchanged (S8A and S8B Fig). This implies that NXF1 plays a less dominant role than TPR in the tumorigenesis of lung cancer. It is possible that overexpression of TPR in lung cancer cells causes mislocalization of its interaction partners such as NXF1, leading to dysregulated nuclear export of RNAs and proteins related to cancer development. Moreover, elevated expression of TPR is not universal to all cancer types. A proteome analysis of six pairs of tumor and normal tissues from colorectal carcinoma patients showed an over fourfold decrease of TPR expression in colorectal cancer biopsies [73]. Our analysis of clinical data suggests that smoking might contribute to elevated expression of TPR (Table F in S1 Text). The underlying mechanism still needs clarification, but is probably associated with changes in DNA methylation status and chromatin remodeling caused by specific tobacco carcinogens [74]. Finally, apart from nuclear export, TPR also participates in transcription regulation, chromatin organization, SUMOylation, mitosis, and telomere length control [75]. Thus, the complete pathway from aberrant expression of TPR to full disease may involve multiple cellular processes and vary in different cancer types.

## Materials and methods

### Ethics statement

Surgically removed lung cancer tissues and adjacent normal tissues were collected from Sun Yat-sen University Cancer Center. The experiments involving the tissue samples were approved by the Research Ethics Committee of Sun Yat-sen University Cancer Center (No. GZR2017-079). Written informed consent was obtained from all subjects, and the experiments conformed to the principles set out in the WMA Declaration of Helsinki and the Department of Health and Human services Belmont Report.

### Yeast strains

All yeast strains used in this study are listed in Table A in S1 Text. Complete ORF deletions and gene fusions were generated by PCR-mediated gene replacement, and the modifications were confirmed by diagnostic PCR products. Yeast strains were cultured in yeast extract-peptone-dextrose (YPD) or synthetic complete (SC) medium at 30°C. M-phase arrest was carried out as described previously [26].

### Cell lines

HLF cells were cultured in Dulbecco’s modified Eagle’s medium (DMEM) supplemented with 10% fetal bovine serum (FBS), while A549, H322, H460 and H1299 cells were cultured in RPMI-1640 medium supplemented with 10% FBS. All cells were maintained at 37°C in a humidified incubator with 5% CO_2_.

### Plasmids

The integrative plasmid YIp-NLS-mCherry (URA3) was generated from the shuttle vector pXRU3 as described in Chou *et al* 2015 [76]. The plasmid contains a cassette that expresses mCherry fused to the nuclear localization signal (NLS) of Nab2. The plasmid was linearized by *Asc*I digestion and integrated into the HO promoter (HOp) of yeast strains to label the nuclei in live cells.

The integrative plasmid pTSK368 (MET25p-MS2-CP-NLS-3×yeGFP::HIS3) containing the MS2 coat protein (MS2-CP) was from addgene (#34551). To generate pTSK368-MET25p-MS2-CP-ΔNLS-1×yeGFP, the NLS was replaced with an oligonucleotide linker (delNLS for/rev in Table B in S1 Text) and the 3×yeGFP was replaced with a PCR product containing a single yeGFP (1×yeGFP for/rev in Table B in S1 Text). The plasmid was integrated into the genome at *his3* after digestion with *Ale*I to yield transformants that were HIS+.

Plasmid pRJG1 was constructed by cloning the *Saccharomyces bayanus tS(CGA)C* gene (Sb-tS(CGA)C) from pMC8 [26] into YCplac111 to generate pMC9. The intron of Sb-tS(CGA)C in pMC9 was first replaced with a single MS2 site and a unique restriction site, which was opened and replaced with a PCR product containing two tandem MS2 sites by homologous recombination in yeast (YCPlac111_F2 and 2×MS2-hp tail rev in Table B in S1 Text). pRJG1 was recovered from yeast, transformed into DH5α competent cells and confirmed by sequencing.

All sequences are available on request.

### Yeast live cell imaging

Slide preparation, cell cycle stage determination and NPC colocalization studies were performed as described previously [26]. For the MS2-GFP studies, fluorescence images were taken with a Zeiss Axioplan II fluorescence microscope (100× objective) with Axiocam HR camera. Z-stacks consisted of 5 planes, each separated by 250 nm, and the acquisition time was 250 miliseconds for each image. Data were analyzed with Image J software. Stacks of the mCherry and GFP channels (nucleus and MS2-GFP, respectively) were collected. In the GFP channel, circular regions of 80 pixels were randomly selected in the nucleus and the cytoplasm. The mean grey value (MGV) for each region was measured, and the ratio of nuclear/cytoplasmic pre-tRNA^Ser(CGA)-2×MS2bs^ distribution was determined as MGV_nucleus_ / MGV_cytoplasm_. All data sets were based on at least three independent trials, totaling to at least 150–250 cells per condition. The trials were then pooled and the data were presented with column scatter graphs. Values for different experimental conditions and strains were compared with one another for statistical significance by Student’s *t*-tests.

### Chromatin immunoprecipitation (ChIP)

ChIP with anti-TAP antibody (Thermo Fisher Scientific CAB1001) and protein A-coated Dynabeads (Thermo Fisher Scientific 10001D) was performed as described previously [26]. Primers used in subsequent qPCR are listed in Table B in S1 Text.

### RNA preparation and RT-qPCR

Yeast RNA was extracted using the hot acidic phenol method, while mammalian RNA was prepared using RNAeasy Animal Total RNA Isolation Kit (Beyotime R0032). For nuclear and cytoplasmic fraction separation, 5×10^6^ mammalian cells were washed twice with ice-cold PBS, collected, resuspended in 500 μL hypotonic buffer (20 mM Tris-HCl pH 7.4, 10 mM NaCl, 3 mM MgCl_2_) by pipetting several times, and incubated on ice for 15 minutes. 25 μL 10% NP-40 was then added, and the homogenate was vortexed for 10 seconds at highest setting before centrifugation at 3,000 rpm at 4°C. The supernatant contained the cytoplasmic fraction, while the pellet was the nuclear fraction.

Reverse transcription was performed using BeyoRT II cDNA First Strand Synthesis Kit (RNase H-) (Beyotime D7168) with gene-specific primers, followed by qPCR using BeyoFast SYBR Green qPCR Mix (2×) (Beyotime D7260) with the primers listed in Table B in S1 Text.

### Northern blot analysis

Nonradioactive Northern blot experiments were performed as described in Wu *et al* 2013 [77] using DIG-labeled probes (Table B in S1 Text), and quantified with Image J software.

### Fluorescence *in situ* hybridization (FISH)

FISH was carried out as described in Sarkar *et al* 1998 [32] using a probe to detect pre-tRNA^Ile(UAU)^ (Table B in S1 Text).

### tRNA microarray assay

Total cellular RNA was prepared with RNAeasy Animal Total RNA Isolation Kit (Beyotime R0032). tRNA demethylation and reverse transcription were performed with rtStar tRNA-optimized First-Strand Synthesis Kit (Arraystar AS-FS-004). qPCR for tRNAs was performed using the nrStar Human tRNA PCR Array (Arraystar AS-NR-001-1) and Arraystar SYBR Green qPCR Master Mix(ROX+) (Arraystar AS-MR-006-5). The data were analyzed by the ΔΔCt method.

### RNA immunoprecipitation (RIP)

RIP was performed following a protocol slightly adapted from Vogt and Taylor 2013 [78]. Briefly, cells were fixed, lysed, sonicated and centrifuged to collect the supernatant, which was then incubated with anti-NXF1 or anti-XPO1 antibody overnight at 4°C with rotation. A sample incubated with IgG was included as control. Dynabeads protein G (Thermo Fisher Scientific 10003D) were added to the antibody conjugated complex and incubated for 2 hours at 4°C with rotation. Afterward, the beads were washed and incubated for 1 hour at 70°C to reverse cross-link. Immunoprecipitated RNA was extracted with RNAeasy Animal Total RNA Isolation Kit (Beyotime R0032), and analyzed by RT-qPCR with the primers listed in Table B in S1 Text.

### RNAi and transfection

siRNA sequences are listed in Table B in S1 Text. siRNA transfection was performed with EndoFectin Max (GeneCopoeia EF013) according to the manufacturer’s instruction.

### Western blot

Antibodies used in this study are rabbit anti-TAP (Thermo Fisher Scientific CAB1001), mouse anti-TPR (abcam ab58344), rabbit anti-eIF2α (Cell Signaling Technology #5324), rabbit anti-phospho-eIF2α(Ser51) (Cell Signaling Technology #3398), normal rabbit IgG (Cell Signaling Technology #2729), rabbit anti-XPO1 (Cell Signaling Technology #46249), rabbit anti-NXF1 (proteintech 10328-1-AP), rabbit anti-NUP62 (proteintech 13916-1-AP), mouse anti-β-tubulin (proteintech 66240-1-Ig), HRP-conjugated affinipure goat anti-mouse IgG(H+L) (proteintech SA00001-1), and HRP-conjugated affinipure goat anti-rabbit IgG(H+L) (proteintech SA00001-2).

Yeast protein extracts were prepared as described in Tsang *et al* 2018 [79]. Mammalian cells were lysed at 4°C with RIPA buffer (Beyotime P0013B) containing protease and phosphatase inhibitors and PMSF, and centrifuged at 12,000 ×*g* for 15 minutes to collect the supernatant. Protein lysates were separated by SDS-polyacrylamide gel electrophoresis (PAGE) and transferred to 0.2 μm PVDF membranes (Roche 03010040001). The membrane was blocked with 5% nonfat milk in PBST for 1 hour at room temperature, incubated with primary antibodies overnight at 4°C, washed three times with PBST, incubated with secondary antibodies for 30 minutes at room temperature, washed three more times with PBST, developed using the enhanced chemiluminescent (ECL) detection reagents, visualized with ChemiDoc Touch Imaging System (Bio-Rad), and quantified with Image J software.

### Cell viability assay

Cell viability assay was performed according to the instruction of CellTiter 96 AQ_ueous_ One Solution Cell Proliferation Assay (MTS) (Promega G3582).

### Colony formation assay

Cells were seeded in six-well plates (800 cells / well) and grown for 7 days. Colonies formed were then fixed with 4% paraformaldehyde for 10 minutes at room temperature, washed twice with H_2_O, stained with crystal violet for 5 minutes, washed with H_2_O for another three times to remove excessive dye, and counted after air-dry overnight.

### FUNCAT

Fluorescent noncanonical amino acid tagging (FUNCAT) was performed as described [80]. Briefly, cells were seeded in 6-cm dishes and grown to 80% confluency. Then, the cells were washed twice with methionine-free DMEM (Gibco 21013024) pre-warmed to 37°C, and incubated in methionine-free DMEM for 30 minutes before the addition of 4 mM azidohomoalanine (AHA) (Sigma 900892), while 4 mM methionine was used as control. For flow cytometry analysis, the cells were collected, fixed in 1% paraformaldehyde in PBS for 15 minutes on ice, and permeabilized in PBS containing 0.1% saponin (Beyotime P0095) for 5 minutes at room temperature. Afterward, Fluor 488-Alkyne (Sigma 761621) was covalently and chemoselectively attached to the introduced azide group by “click chemistry”. Cells were then washed twice and resuspended in PBS. Fluor 488 signal was detected by a CytoFLEX flow cytometer (Beckman Coulter) and analyzed by CytExpert 2.4 software.

### Cell cycle analysis

Cells were harvested, fixed in pre-chilled 70% ethanol at 4°C overnight, washed twice with PBS, and incubated in PI/RNase staining buffer (BD 550825) at room temperature for 15 minutes. Cell numbers in different cell cycle stages were counted by CytoFLEX benchtop flow cytometer (Beckman Coulter), and the data were analyzed by ModFit LT 4.1 software.

## Supporting information

S1 Fig Characterization of *tS(CGA)C-2×MS2bs*. (A) Meiotic segregation of markers among tetrads bearing *tS(CGA)C*-*2×MS2bs*. Left panels: Tetrads from a diploid strain [*MATa*/*MATα Δts(cga)c*::*ble*^*r*^/*tS(CGA)C Δlos1*::*hphMX* / *LOS1 his3Δ1*/*his3Δ1*::*TDH3p-MS2CP-yeGFP*::*HIS3* with plasmid pRJG1 expressing *tS(CGA)C*-*2×MS2bs*::*LEU2*] were spotted on different selective plates: YPDA (yeast extract + peptone + dextrose + adenine, as loading control), SC-leu [synthetic complete medium lacking leucine to select for the plasmid with *tS(CGA)C*-*2×MS2bs*::*LEU2*], YPDA+zeo [YPDA medium containing zeocin to select for *Δts(cga)c*::*ble*^*r*^], YPDA+hygro (YPDA medium containing hygromycin to select for *Δlos1*::*hphMX*), and SC-his (synthetic complete medium lacking histidine to select for *TDH3p-MS2CP-yeGFP*::*HIS3*) plates. Tetrad H1 was saved as MRG 5789. Right panel: Fivefold serial dilutions of tetrads J4 [*tS(CGA)C*], G3 [*tS(CGA)C* + *Δlos1*], I3 [*tS(CGA)C*-*2×MS2bs*] and H1 [*tS(CGA)C*-*2×MS2bs* + *Δlos1*] were spotted on synthetic complete medium to compare their growth rate. *tS(CGA)C*-*2×MS2bs* provided the only source of the essential CGA-codon tRNA to the cell in I3 and H1, and their growth was largely unchanged. (B) A single 3′-DIG labeled LNA-modified oligonucleotide probe was used to detect pre-tRNA^Ser(CGA)-2×MS2bs^ and mature tRNA^Ser(CGA)^, as well as pre-tRNA^Ser(CGA)^. An uncropped, 2-minute exposure is shown on the left. A 5-minute exposure of a second gel loaded with higher levels of RNA extracts is shown on the right. (C) A different oligonucleotide probe was used to detect both pre-tRNA^Ser(CGA)-2×MS2bs^ (pre) and the spliced 2×MS2bs intron within the same sample preparations of Fig 2D. A 2-minute exposure of the pre-tRNA and a 5-minute exposure of the spliced intron are shown. The ratio of pre / intron is indicated below each lane.
(TIF)

S2 Fig Live cell imaging of nascent tRNA nuclear export in yeast. Representative images of cells expressing MS2-GFP and pre-tRNA^Ser(CGA)^ with (+MS2bs) or without MS2 sites (-MS2bs). Nab2-mCherry shows the nuclei. Imaged were strains MRG5788 (*wt*), MRG5789 (*Δlos1*), MRG5817 (*Δmlp1*,*2*), and their derivatives with either pJW031-LYS2[*tS(CGA)C/LYS2/CEN*] or pRJG1[YCplac111-*Sb-tS(CGA)C-2×MS2bs*::*LEU2*]. Scale bar = 10 μm.
(TIF)

S3 Fig TPR expression in lung cancer. (A) Disease summary for TPR from ONCOMINE. Cell color represented the best gene rank percentile for the analyses included in the cell. (B) TPR protein expression in the NCI-60 cell lines grouped according to their tissue origins. Data were obtained from NCI-60 proteome resource, which included a core cancer proteome of 5,578 proteins consistently quantified across various tissue types. (C) Left and Middle: TPR mRNA level in the NCI-60 cell lines grouped according to their tissue origins. Data were extracted from the GSE5720 (top row) and GSE5846 (bottom row) mRNA expression profiles of the NCI-60 cancer cell panel. 201730_s_at (left) and 201731_s_at (middle) were two different probes used to detect the mRNA level of TPR. Right: Correlation analysis of TPR protein abundance (from NCI-60 proteome resource) and mRNA level in NCI-60 cell lines.
(TIF)

S4 Fig Analyses of protein expression and cell cycle progression in TPR knockdown cells. (A) Quantification of the western blot results in control and TPR knockdown (Tsi, Tsi-1 and Tsi-2) A549, H322, H460 and H1299 cells. TPR / β-tubulin ratios (mean ± SD, n = 3) were analyzed by paired Student’s *t*-test; *P < 0.05. (B) FACS profiles and quantifications of lung cancer cells. A549, H322, H460 and H1299 cells were treated with control (ctrl) or TPR siRNAs (Tsi-1 and Tsi-2), fixed with 70% ethanol and stained with PI before analyzed by FACS for ploidy. The vertical axis indicates cell number, while the horizontal axis is for DNA content. G1 and G2 phases are respectively represented as the first and second peaks starting from the vertical axis. S phase is marked as the intermediate striped domain. Each trial included at least 10,000 cells, and data (mean ± SD, n = 2) were analyzed by paired student’s *t*-test.
(TIF)

S5 Fig Nuclear accumulation of tRNAs in TPR knockdown cells. (A) Correlation analysis of relative abundance of tRNAs in Tsi and control cells. (B) Relative nuclear enrichment of tRNAs in H322 (top) and H1299 (bottom) cells. Nuclear and whole cell RNA extracts were prepared from control cells and cells with TPR knockdown (Tsi, Tsi-1 and Tsi-2). RT-qPCR was performed for each extract to measure the level of tRNA^Glu(CTC)^, tRNA^Tyr(GTA)^, tRNA^His(GTG)^, tRNA^Leu(CAG)^, tRNA^Gly(CCC)^ and tRNA^Gly(GCC)^, using U6 as internal control. Relative nuclear enrichment of each tRNA was determined by its nuclear / total expression normalized to the same ratio of the tRNA within control. Data were presented as mean ± SD (n = 2).
(TIF)

S6 Fig tRNA levels in A549 cells with TPR knockdown. (A) Total abundance of tRNAs. Whole cell RNA extracts were prepared from control cells and cells with TPR knockdown (Tsi, Tsi-1 and Tsi-2). RT-qPCR was performed to measure the total abundance of tRNA^Glu(CTC)^, tRNA^Tyr(GTA)^, tRNA^His(GTG)^, tRNA^Leu(CAG)^, tRNA^Gly(CCC)^ and tRNA^Gly(GCC)^, using U6 as internal control. Data were normalized to the control and presented as mean ± SD (n ≥ 2). (B) Nascent pre-tRNA levels. Whole cell RNA extracts were prepared from control cells and cells with TPR knockdown (Tsi, Tsi-1 and Tsi-2). RT-qPCR was performed to measure the levels of nascently transcribed pre-tRNA^Arg(TCT)^, pre-tRNA^Tyr(GTA)^ and pre-tRNA^Leu(CAA)^, using U6 as internal control. Data were normalized to the control and presented as mean ± SD (n = 2).
(TIF)

S7 Fig Nuclear accumulation of tRNAs and reduced growth in NXF1 knockdown cells. (A) Nuclear enrichment of tRNAs in H322 (top), H460 (middle) and H1299 (bottom) cells without (control) and with NXF1 knockdown (Nsi-1 and Nsi-2). Relative nuclear enrichment of each tRNA tested was determined by its nuclear / total expression normalized to the same ratio of the tRNA within the control, shown as mean ± SD (n = 2), and analyzed by Student’s *t*-test; *P < 0.05. (B) Quantification of colony formation assay done in control and NXF1 knockdown (Nsi-1 and Nsi-2) cells. Data (mean ± SD, n = 3) were analyzed by paired student’s *t*-test; **P < 0.01.
(TIF)

S8 Fig NXF1 expression in patient tissues and TPR knockdown cells. (A) NXF1 mRNA level in human lung cancer (T) and adjacent non-tumor (N) tissues. Data were extracted from GSE7670, GSE10072 and GSE31210, normalized to the median value of the non-tumor tissues in each dataset, and compiled for analysis. (B) Left: Western blot to detect the expression of NXF1 in paired tumor (T) and adjacent non-tumor (N) tissues from lung cancer patients. β-tubulin served as the loading control. Right: Quantification of NXF1 expression relative to β-tubulin in paired tumor (T) and adjacent non-tumor (N) tissues from lung cancer patients. (C) Western blot to detect the expression of NXF1 in control and TPR knockdown (Tsi, Tsi-1 and Tsi-2) A549, H322, H460 and H1299 cells. β-tubulin was used as the loading control.
(TIF)

S9 Fig Inhibited protein synthesis in TPR knockdown cells. (A) Western blot to detect the phosphorylation of eIF2α. Protein extracts were prepared from H322 and H460 cells without (control) or with TPR knockdown (Tsi, Tsi-1 and Tsi-2). Numbers indicate the ratios of TPR / β-tubulin (top) and p-eIF2α / eIF2α (bottom). (B-C) Correlation of cytoplasmic abundance of different tRNA species (B), tRNA^Pro^ (C top) and tRNA^Ser^ (C bottom) isoacceptors in control (ctrl) and TPR knockdown (Tsi) A549 cells to codon usage of the top 10 up-regulated (Up) and the 10 most down-regulated (Down) genes in lung cancer tissues with high TPR expression. Cytoplasmic abundance of tRNA was calculated as tRNA_cyt_ = tRNA_tot_ - tRNA_nuc_, using the data from the tRNA PCR array.
(TIF)

S10 Fig Nuclear accumulation of other RNAs in A549 cells with TPR knockdown. (A) Measurement of nuclear RNA enrichment. Nuclear and whole cell RNA extracts were prepared from A549 cells (control) and A549 cells with TPR knockdown (Tsi, Tsi-1 and Tsi-2). RT-qPCR was performed for each extract to measure the level of U1 snRNA, GAPDH mRNA and 5.8S rRNA, using U6 as an internal control. Relative nuclear enrichment of each RNA was determined by its nuclear / total expression normalized to the ratio within the control. Data were presented as mean ± SD (n = 2). (B) Total abundance of other RNAs. Whole cell RNA extracts were prepared from control cells and cells with TPR knockdown (Tsi, Tsi-1 and Tsi-2). RT-qPCR was performed to measure the total abundance of U1 snRNA, GAPDH mRNA and 5.8S rRNA, using U6 as internal control. Data were normalized to the control and presented as mean ± SD (n = 2).
(TIF)

S11 Fig Expression of proteins related to NPC functions in cells with TPR knockdown. Western blot to detect the expression of TPR, XPO1, NXF1 and NUP62 in A549 cells transfected with TPR-specific siRNAs (Tsi-1 and Tsi-2) or a control siRNA.
(TIF)

S1 Text Supplementary tables. (A) Yeast strains. (B) Oligonucleotides. (C) TPR mRNA level in the NCI-60 cell lines from GSE5720. (D) TPR mRNA level in the NCI-60 cell lines from GSE5846. (E) TPR protein abundance in cell lines from the NCI-60 proteome resource. (F) Correlation analysis of TPR mRNA level and clinicopathological features in lung cancer patients.
(DOCX)

S1 Data Numerical data for graphs in main figures. (XLSX)

S2 Data Numerical data for graphs in supplementary figures. (XLSX)
