# Supplementary figures and images for: Nucleoporin TPR promotes tRNA nuclear export and protein synthesis in lung cancer cells
Source: PLoS Genet. 2021 Nov 18;17(11):e1009899. doi: 10.1371/journal.pgen.1009899 (PMC8639082; doi:10.1371/journal.pgen.1009899)

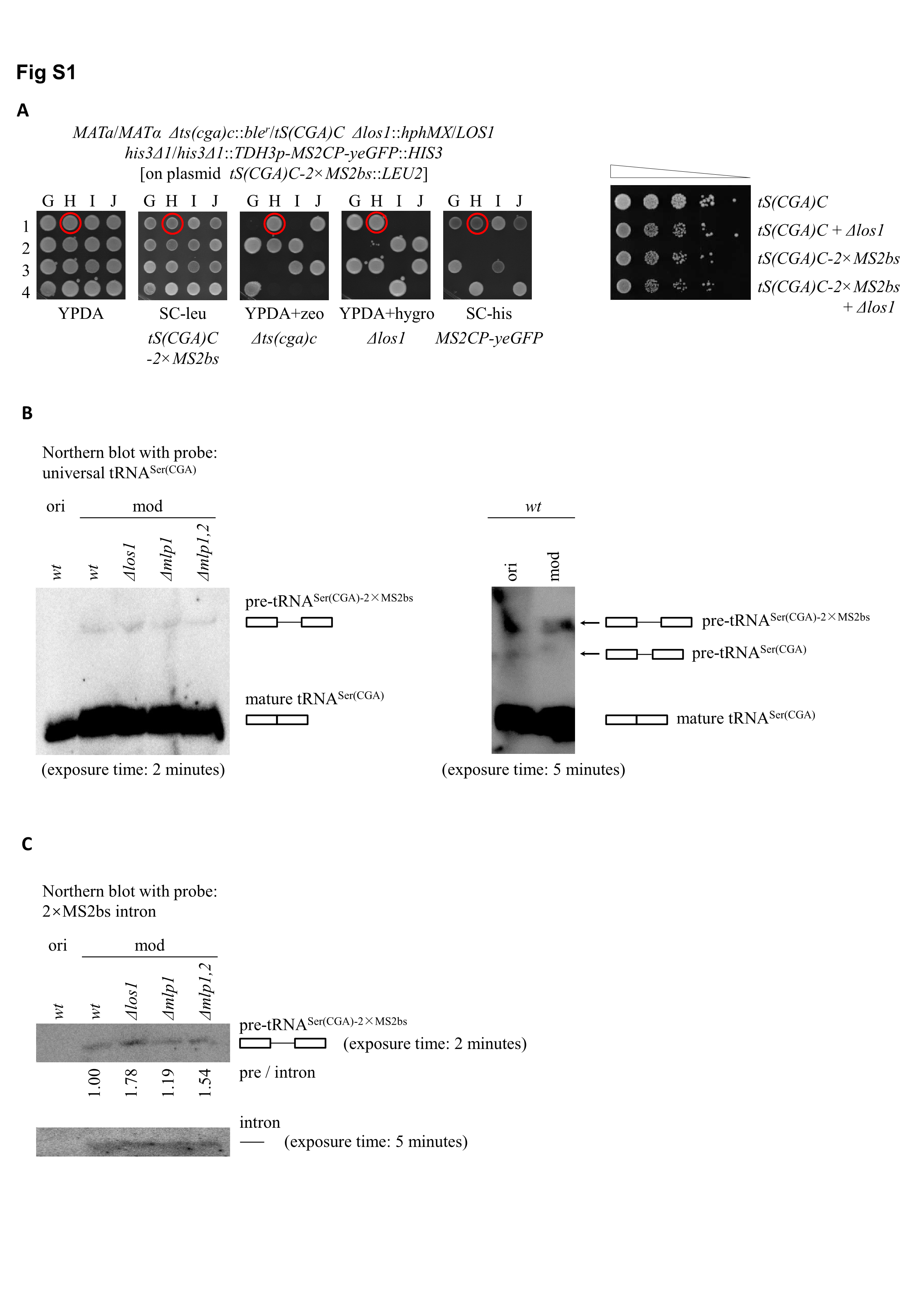

Supplement: S1 Fig — (A) Meiotic segregation of markers among tetrads bearing tS(CGA)C-2×MS2bs. Left panels: Tetrads from a diploid strain [MATa/MATα Δts(cga)c::bler/tS(CGA)C Δlos1::hphMX / LOS1 his3Δ1/his3Δ1::TDH3p-MS2CP-yeGFP::HIS3 with plasmid pRJG1 expressing tS(CGA)C-2×MS2bs::LEU2] were spotted on different selective plates: YPDA (yeast extract + peptone + dextrose + adenine, as loading control), SC-leu [synthetic complete medium lacking leucine to select for the plasmid with tS(CGA)C-2×MS2bs::LEU2], YPDA+zeo [YPDA medium containing zeocin to select for Δts(cga)c::bler], YPDA+hygro (YPDA medium containing hygromycin to select for Δlos1::hphMX), and SC-his (synthetic complete medium lacking histidine to select for TDH3p-MS2CP-yeGFP::HIS3) plates. Tetrad H1 was saved as MRG 5789. Right panel: Fivefold serial dilutions of tetrads J4 [tS(CGA)C], G3 [tS(CGA)C + Δlos1], I3 [tS(CGA)C-2×MS2bs] and H1 [tS(CGA)C-2×MS2bs + Δlos1] were spotted on synthetic complete medium to compare their growth rate. tS(CGA)C-2×MS2bs provided the only source of the essential CGA-codon tRNA to the cell in I3 and H1, and their growth was largely unchanged. (B) A single 3′-DIG labeled LNA-modified oligonucleotide probe was used to detect pre-tRNASer(CGA)-2×MS2bs and mature tRNASer(CGA), as well as pre-tRNASer(CGA). An uncropped, 2-minute exposure is shown on the left. A 5-minute exposure of a second gel loaded with higher levels of RNA extracts is shown on the right. (C) A different oligonucleotide probe was used to detect both pre-tRNASer(CGA)-2×MS2bs (pre) and the spliced 2×MS2bs intron within the same sample preparations of Fig 2D. A 2-minute exposure of the pre-tRNA and a 5-minute exposure of the spliced intron are shown. The ratio of pre / intron is indicated below each lane. (TIF) [file pgen.1009899.s001.tif]

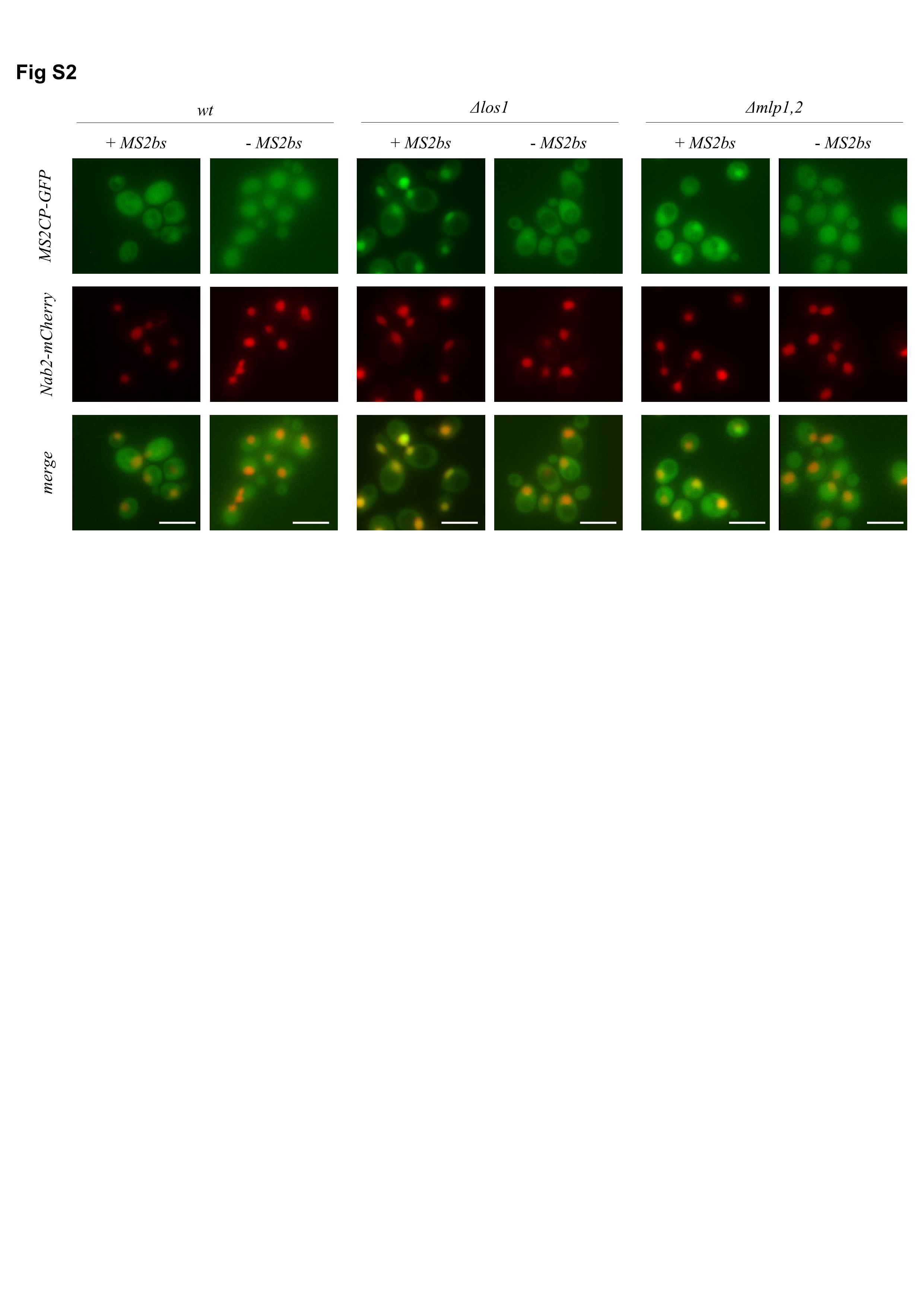

Supplement: S2 Fig — Representative images of cells expressing MS2-GFP and pre-tRNASer(CGA) with (+MS2bs) or without MS2 sites (-MS2bs). Nab2-mCherry shows the nuclei. Imaged were strains MRG5788 (wt), MRG5789 (Δlos1), MRG5817 (Δmlp1,2), and their derivatives with either pJW031-LYS2[tS(CGA)C/LYS2/CEN] or pRJG1[YCplac111-Sb-tS(CGA)C-2×MS2bs::LEU2]. Scale bar = 10 μm. (TIF) [file pgen.1009899.s002.tif]

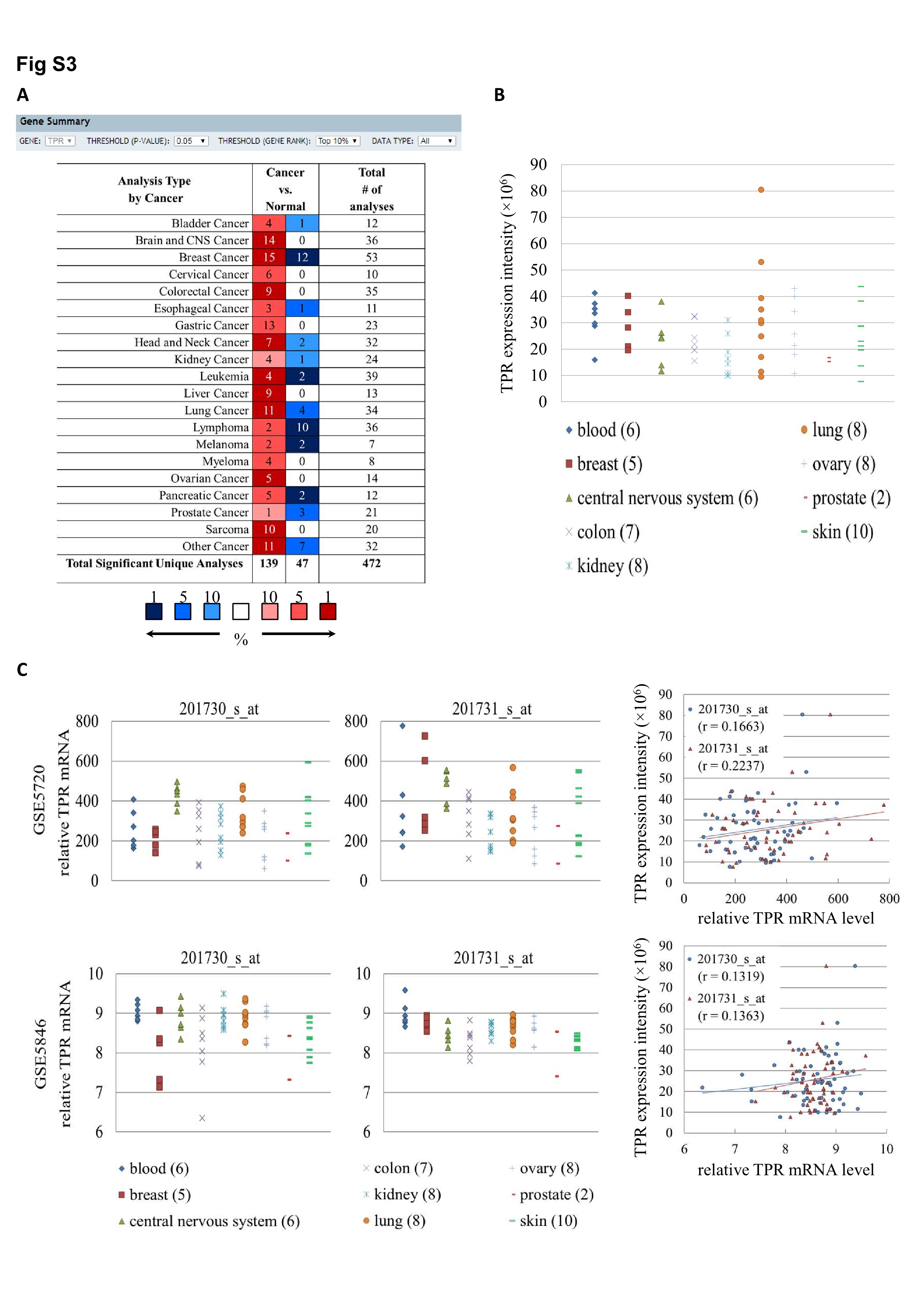

Supplement: S3 Fig — (A) Disease summary for TPR from ONCOMINE. Cell color represented the best gene rank percentile for the analyses included in the cell. (B) TPR protein expression in the NCI-60 cell lines grouped according to their tissue origins. Data were obtained from NCI-60 proteome resource, which included a core cancer proteome of 5,578 proteins consistently quantified across various tissue types. (C) Left and Middle: TPR mRNA level in the NCI-60 cell lines grouped according to their tissue origins. Data were extracted from the GSE5720 (top row) and GSE5846 (bottom row) mRNA expression profiles of the NCI-60 cancer cell panel. 201730_s_at (left) and 201731_s_at (middle) were two different probes used to detect the mRNA level of TPR. Right: Correlation analysis of TPR protein abundance (from NCI-60 proteome resource) and mRNA level in NCI-60 cell lines. (TIF) [file pgen.1009899.s003.tif]

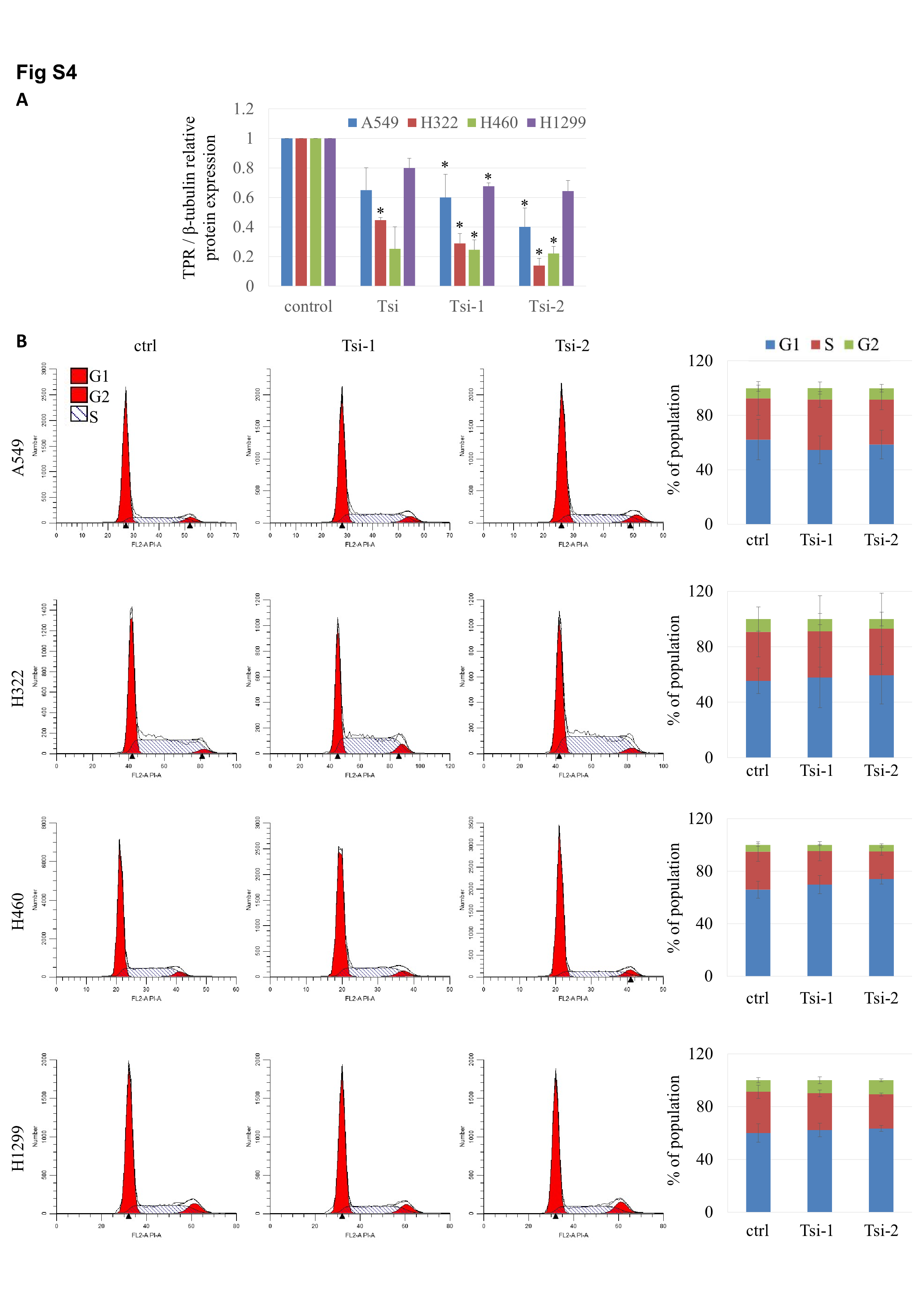

Supplement: S4 Fig — (A) Quantification of the western blot results in control and TPR knockdown (Tsi, Tsi-1 and Tsi-2) A549, H322, H460 and H1299 cells. TPR / β-tubulin ratios (mean ± SD, n = 3) were analyzed by paired Student’s t-test; *P < 0.05. (B) FACS profiles and quantifications of lung cancer cells. A549, H322, H460 and H1299 cells were treated with control (ctrl) or TPR siRNAs (Tsi-1 and Tsi-2), fixed with 70% ethanol and stained with PI before analyzed by FACS for ploidy. The vertical axis indicates cell number, while the horizontal axis is for DNA content. G1 and G2 phases are respectively represented as the first and second peaks starting from the vertical axis. S phase is marked as the intermediate striped domain. Each trial included at least 10,000 cells, and data (mean ± SD, n = 2) were analyzed by paired student’s t-test. (TIF) [file pgen.1009899.s004.tif]

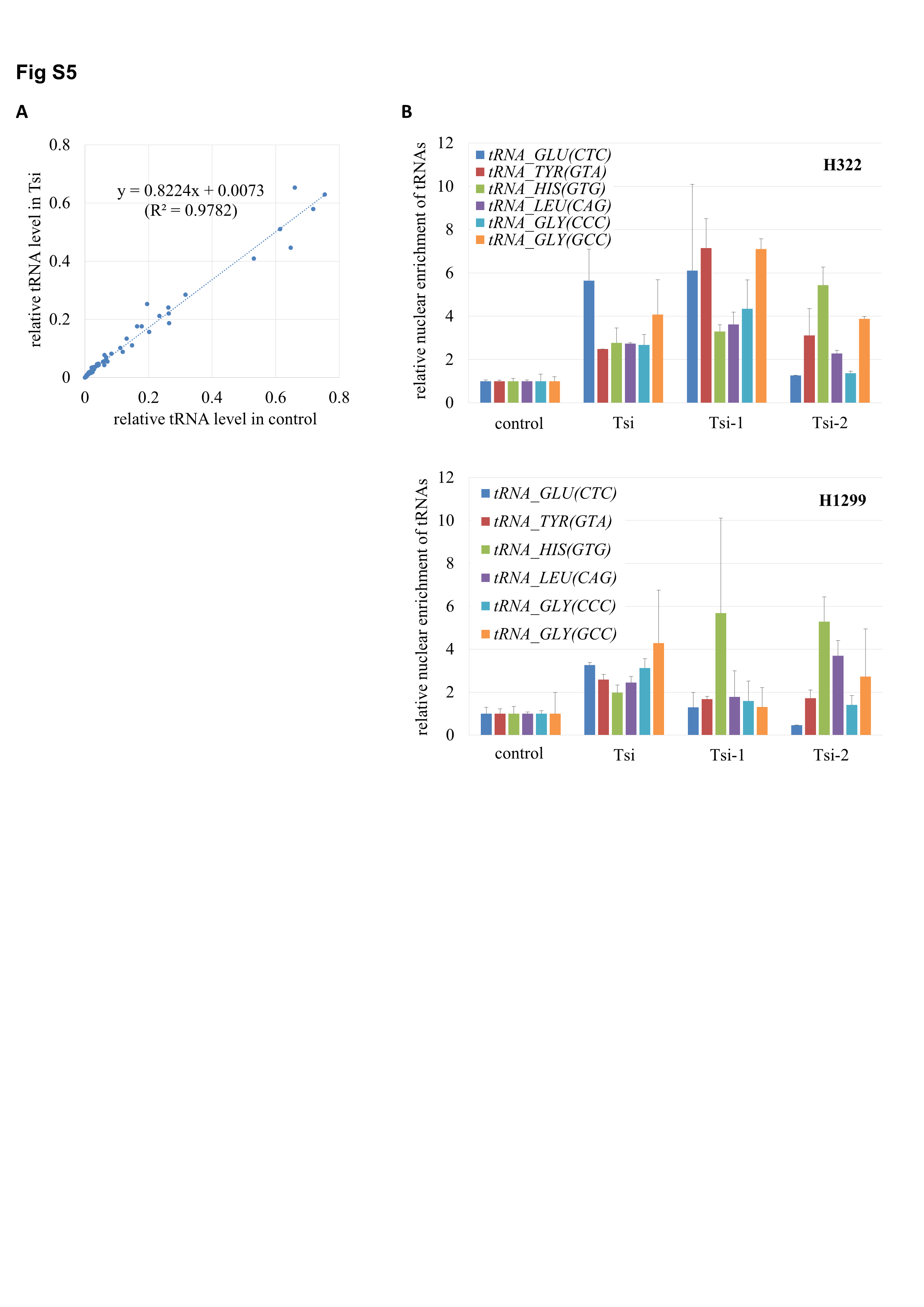

Supplement: S5 Fig — (A) Correlation analysis of relative abundance of tRNAs in Tsi and control cells. (B) Relative nuclear enrichment of tRNAs in H322 (top) and H1299 (bottom) cells. Nuclear and whole cell RNA extracts were prepared from control cells and cells with TPR knockdown (Tsi, Tsi-1 and Tsi-2). RT-qPCR was performed for each extract to measure the level of tRNAGlu(CTC), tRNATyr(GTA), tRNAHis(GTG), tRNALeu(CAG), tRNAGly(CCC) and tRNAGly(GCC), using U6 as internal control. Relative nuclear enrichment of each tRNA was determined by its nuclear / total expression normalized to the same ratio of the tRNA within control. Data were presented as mean ± SD (n = 2). (TIF) [file pgen.1009899.s005.tif]

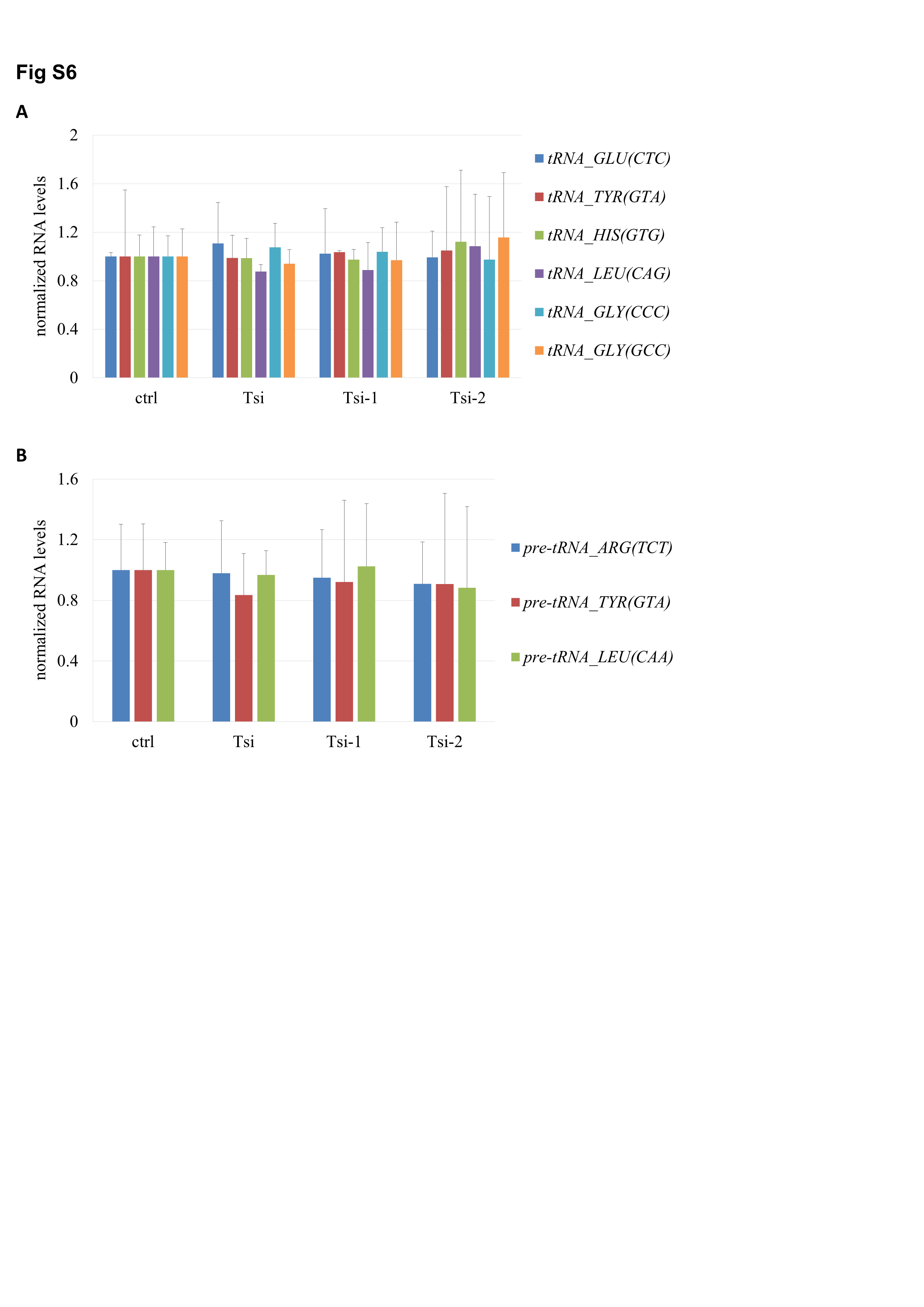

Supplement: S6 Fig — (A) Total abundance of tRNAs. Whole cell RNA extracts were prepared from control cells and cells with TPR knockdown (Tsi, Tsi-1 and Tsi-2). RT-qPCR was performed to measure the total abundance of tRNAGlu(CTC), tRNATyr(GTA), tRNAHis(GTG), tRNALeu(CAG), tRNAGly(CCC) and tRNAGly(GCC), using U6 as internal control. Data were normalized to the control and presented as mean ± SD (n ≥ 2). (B) Nascent pre-tRNA levels. Whole cell RNA extracts were prepared from control cells and cells with TPR knockdown (Tsi, Tsi-1 and Tsi-2). RT-qPCR was performed to measure the levels of nascently transcribed pre-tRNAArg(TCT), pre-tRNATyr(GTA) and pre-tRNALeu(CAA), using U6 as internal control. Data were normalized to the control and presented as mean ± SD (n = 2). (TIF) [file pgen.1009899.s006.tif]

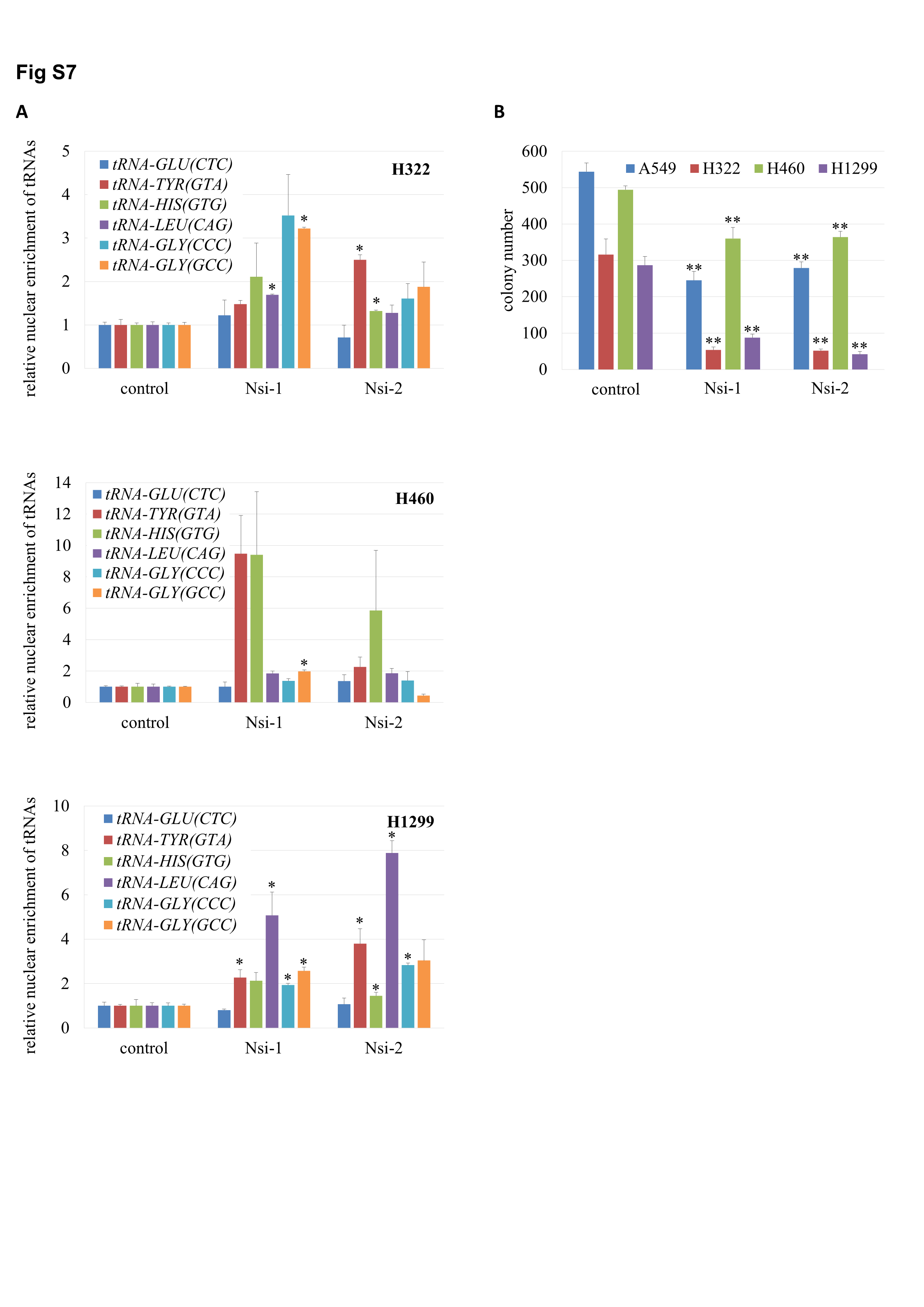

Supplement: S7 Fig — (A) Nuclear enrichment of tRNAs in H322 (top), H460 (middle) and H1299 (bottom) cells without (control) and with NXF1 knockdown (Nsi-1 and Nsi-2). Relative nuclear enrichment of each tRNA tested was determined by its nuclear / total expression normalized to the same ratio of the tRNA within the control, shown as mean ± SD (n = 2), and analyzed by Student’s t-test; *P < 0.05. (B) Quantification of colony formation assay done in control and NXF1 knockdown (Nsi-1 and Nsi-2) cells. Data (mean ± SD, n = 3) were analyzed by paired student’s t-test; **P < 0.01. (TIF) [file pgen.1009899.s007.tif]

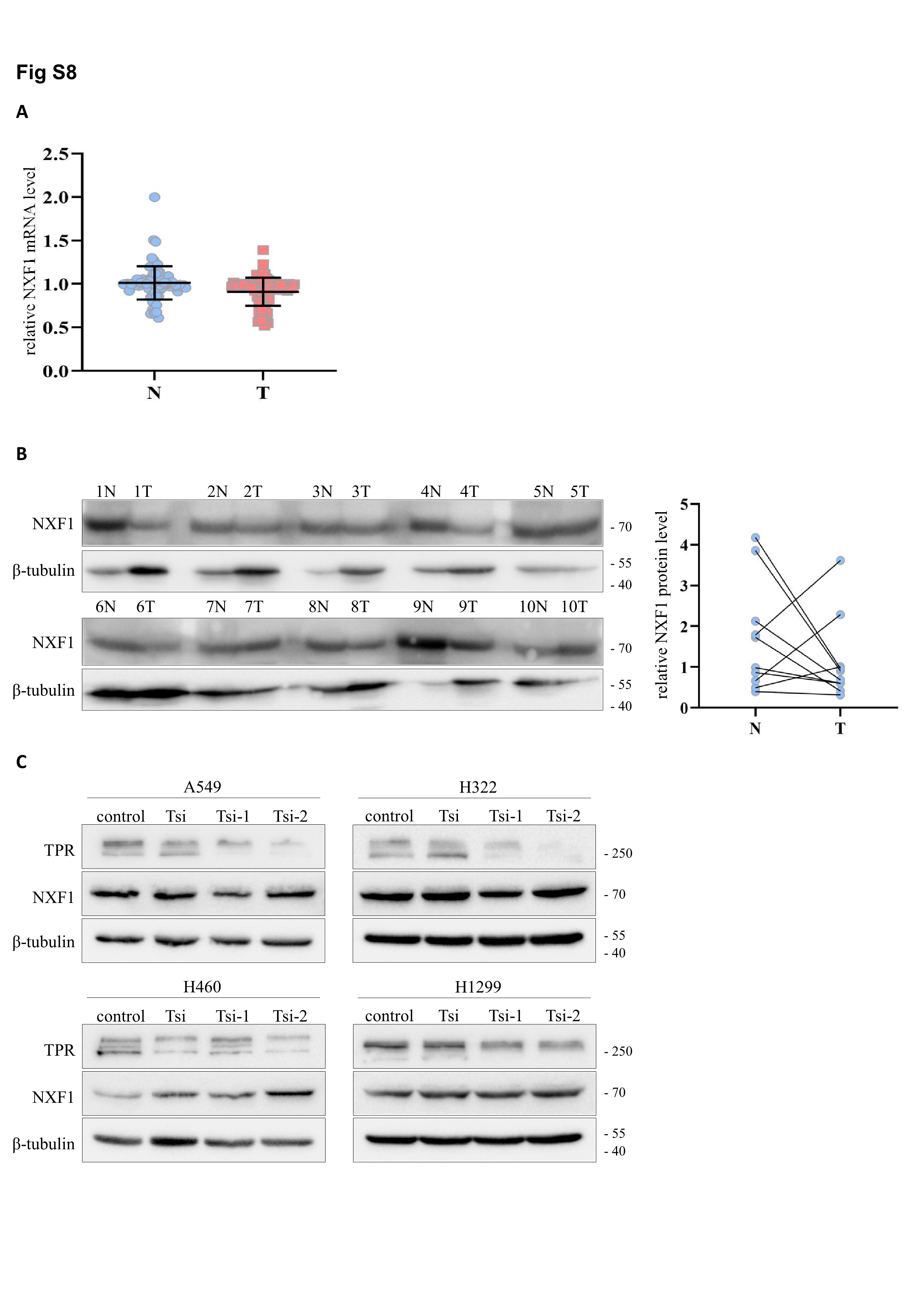

Supplement: S8 Fig — (A) NXF1 mRNA level in human lung cancer (T) and adjacent non-tumor (N) tissues. Data were extracted from GSE7670, GSE10072 and GSE31210, normalized to the median value of the non-tumor tissues in each dataset, and compiled for analysis. (B) Left: Western blot to detect the expression of NXF1 in paired tumor (T) and adjacent non-tumor (N) tissues from lung cancer patients. β-tubulin served as the loading control. Right: Quantification of NXF1 expression relative to β-tubulin in paired tumor (T) and adjacent non-tumor (N) tissues from lung cancer patients. (C) Western blot to detect the expression of NXF1 in control and TPR knockdown (Tsi, Tsi-1 and Tsi-2) A549, H322, H460 and H1299 cells. β-tubulin was used as the loading control. (TIF) [file pgen.1009899.s008.tif]

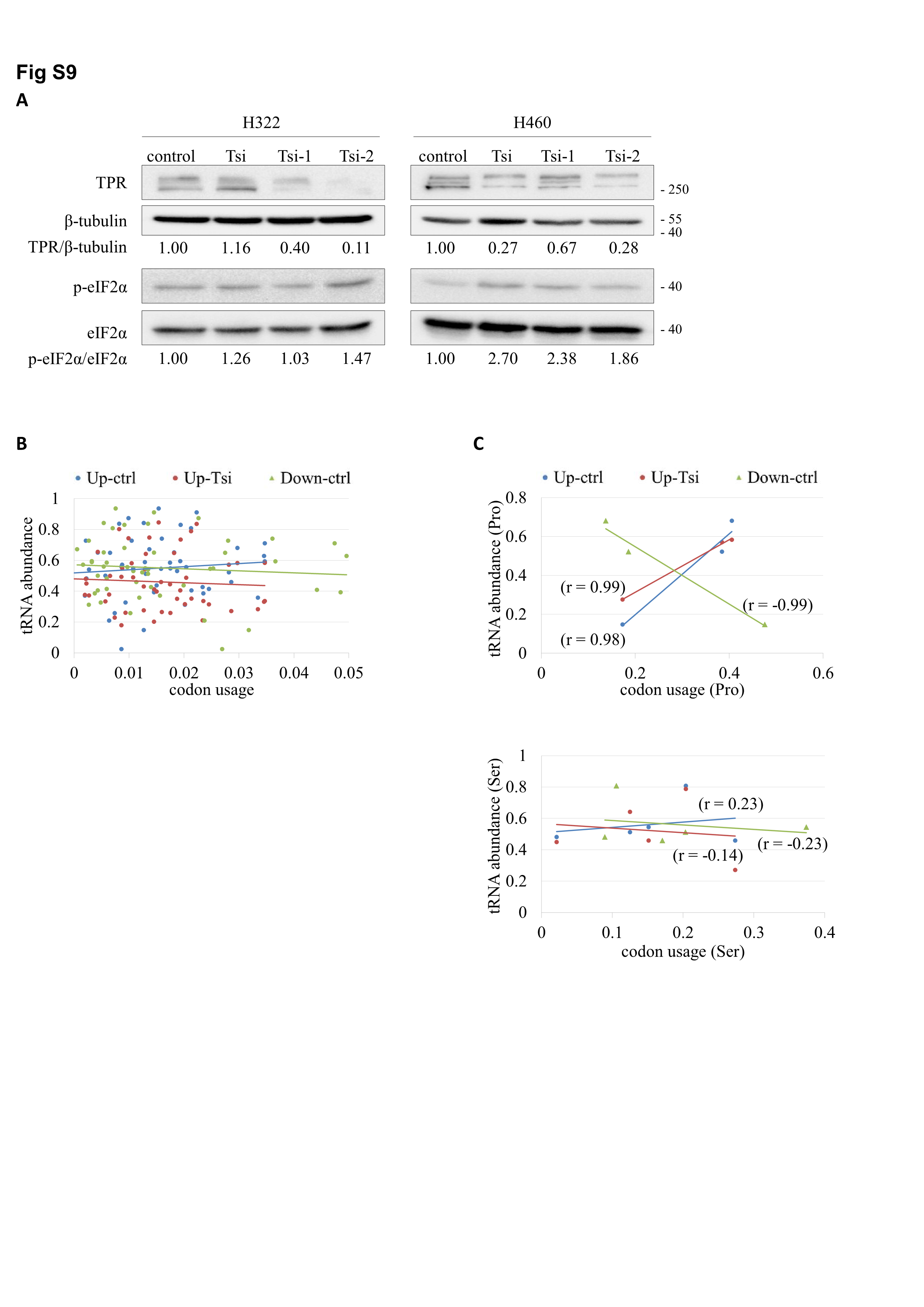

Supplement: S9 Fig — (A) Western blot to detect the phosphorylation of eIF2α. Protein extracts were prepared from H322 and H460 cells without (control) or with TPR knockdown (Tsi, Tsi-1 and Tsi-2). Numbers indicate the ratios of TPR / β-tubulin (top) and p-eIF2α / eIF2α (bottom). (B-C) Correlation of cytoplasmic abundance of different tRNA species (B), tRNAPro (C top) and tRNASer (C bottom) isoacceptors in control (ctrl) and TPR knockdown (Tsi) A549 cells to codon usage of the top 10 up-regulated (Up) and the 10 most down-regulated (Down) genes in lung cancer tissues with high TPR expression. Cytoplasmic abundance of tRNA was calculated as tRNAcyt = tRNAtot - tRNAnuc, using the data from the tRNA PCR array. (TIF) [file pgen.1009899.s009.tif]

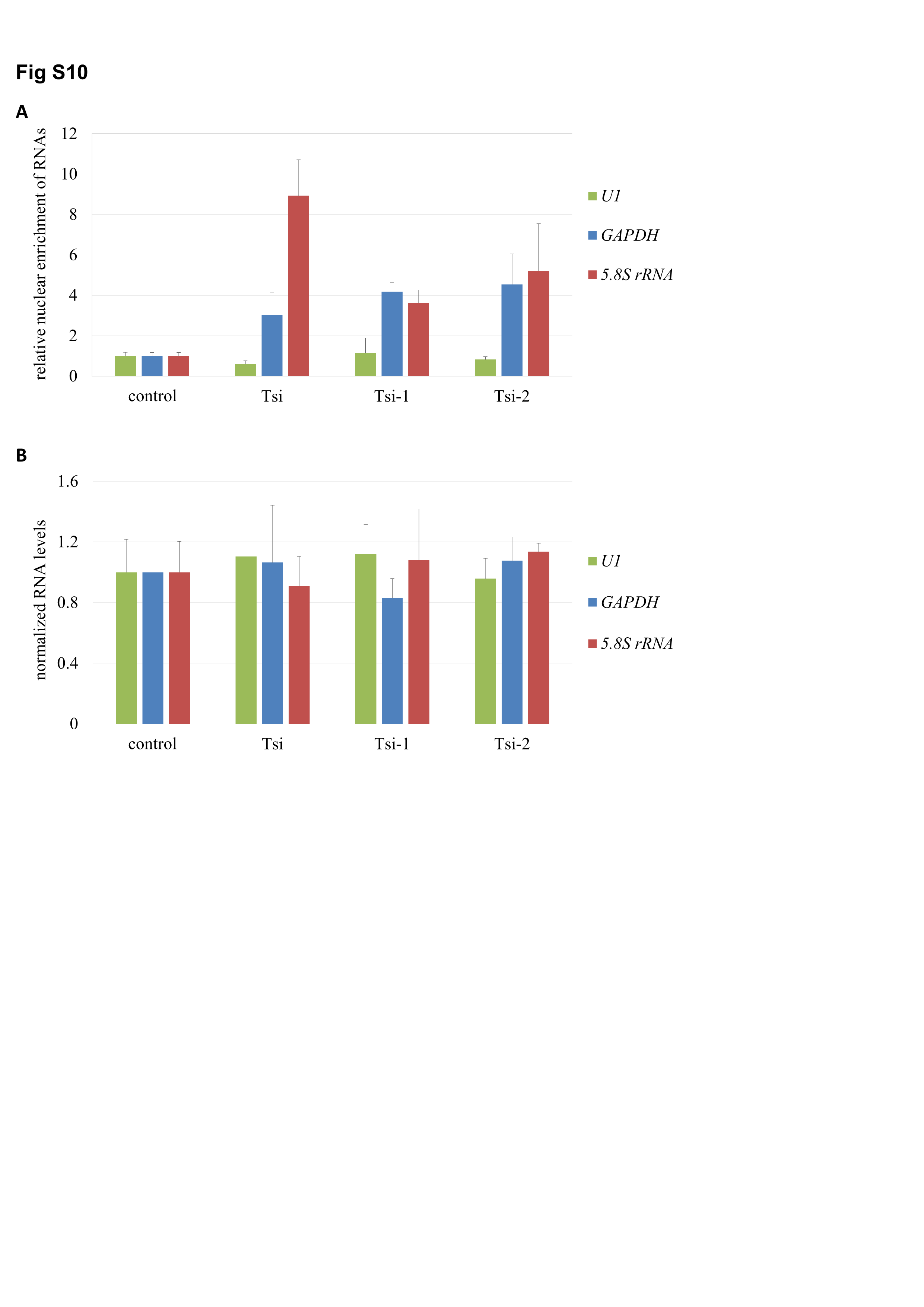

Supplement: S10 Fig — (A) Measurement of nuclear RNA enrichment. Nuclear and whole cell RNA extracts were prepared from A549 cells (control) and A549 cells with TPR knockdown (Tsi, Tsi-1 and Tsi-2). RT-qPCR was performed for each extract to measure the level of U1 snRNA, GAPDH mRNA and 5.8S rRNA, using U6 as an internal control. Relative nuclear enrichment of each RNA was determined by its nuclear / total expression normalized to the ratio within the control. Data were presented as mean ± SD (n = 2). (B) Total abundance of other RNAs. Whole cell RNA extracts were prepared from control cells and cells with TPR knockdown (Tsi, Tsi-1 and Tsi-2). RT-qPCR was performed to measure the total abundance of U1 snRNA, GAPDH mRNA and 5.8S rRNA, using U6 as internal control. Data were normalized to the control and presented as mean ± SD (n = 2). (TIF) [file pgen.1009899.s010.tif]

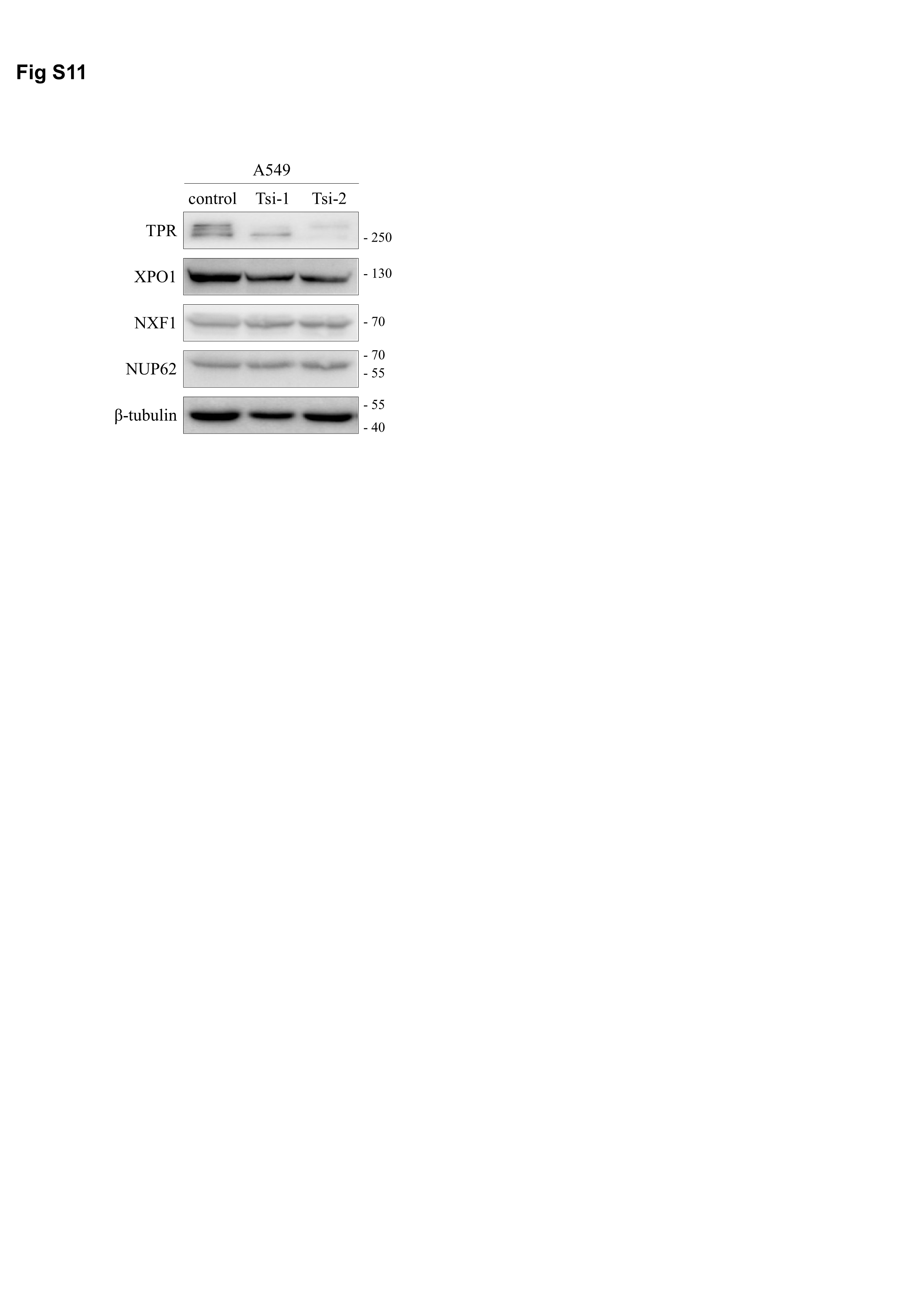

Supplement: S11 Fig — Western blot to detect the expression of TPR, XPO1, NXF1 and NUP62 in A549 cells transfected with TPR-specific siRNAs (Tsi-1 and Tsi-2) or a control siRNA. (TIF) [file pgen.1009899.s011.tif]
