# Supplementary material for: Nucleoporin TPR promotes tRNA nuclear export and protein synthesis in lung cancer cells
Source: PLoS Genet. 2021 Nov 18;17(11):e1009899. doi: 10.1371/journal.pgen.1009899 (PMC8639082; doi:10.1371/journal.pgen.1009899)
Supplement: S1 Text — (A) Yeast strains. (B) Oligonucleotides. (C) TPR mRNA level in the NCI-60 cell lines from GSE5720. (D) TPR mRNA level in the NCI-60 cell lines from GSE5846. (E) TPR protein abundance in cell lines from the NCI-60 proteome resource. (F) Correlation analysis of TPR mRNA level and clinicopathological features in lung cancer patients. (DOCX) [file pgen.1009899.s012.docx]

**Table A** – Yeast strains

| Name | Genotype | Source |
| --- | --- | --- |
| W303-1A | *MAT***a** *ade2-1 can1-100 his3-11,15 leu2-3,112 trp1-1 ura3-1* | [1] |
| BY4741 | *MAT***a** *his3Δ1 leu2Δ0 met15Δ0 ura3Δ0* | [2] |
| BY4742 | *MAT***α** *his3Δ1 leu2Δ0 lys2Δ0 ura3Δ0* | “” |
| MC172 | W303-1A *MET3p-CDC20*::*kanMX* | [3] |
| MC177 | W303-1A *MET3p-CDC20*::*kanMX NUP60-TAP*::*HIS3MX6* | “” |
| MC199 | *MAT***α** *RS*::*HMRE-a2a1-HMRI-GIT1*::*RS* *(LEU2*::*GAL1p-R)2*::*leu2-3,112 ADE2*::*HIS3p-GFPlacI*::*ade2-1* *Δlys2* *NIC96-mRFP*::*natMX Δnup133*::*hphMX tS(CGA)C-BUD31*::*256lac^op^-TRP1 MET3p-CDC20-kanMX [pMC3 (nup133-ΔN)]* | This study |
| MC200 | *MAT***α** *RS*::*HMRE-a2a1-HMRI-GIT1*::*RS* *(LEU2*::*GAL1p-R)2*::*leu2-3,112 ADE2*::*HIS3p-GFPlacI*::*ade2-1* *Δlys2* *NIC96-mRFP*::*natMX Δnup133*::*hphMX tT(UGU)G1*::*256lac^op^-TRP1 MET3p-CDC20-kanMX [pMC3 (nup133-ΔN)]* | “” |
| MC273 | W303-1A *MET3p-CDC20*::*hphMX NUP60-TAP*::*HIS3MX6 Δmlp1*::*kanMX* | “” |
| MC276 | W303-1A *MET3p-CDC20*::*hphMX NUP60-TAP*::*HIS3MX6 Δmlp1*::*TRP1 Δmlp2*::*kanMX* | “” |
| MC341 | BY4742 *GCN4-TAP*::*HIS3MX6* | “” |
| MC343 | BY4741 *GCN4-TAP*::*HIS3MX6 Δlos1*::*hphMX* | “” |
| MC344 | BY4741 or BY4742 *GCN4-TAP*::*HIS3MX6 Δmlp1*::*kanMX* | “” |
| MC345 | BY4741 or BY4742 *GCN4-TAP*::*HIS3MX6 Δmlp1*::*kanMX Δmlp2*::*hphMX* | “” |
| MC349 | BY4741 *GCN4-TAP*::*HIS3MX6 Δnup2*::*hphMX* | “” |
| MC350 | BY4741 *GCN4-TAP*::*HIS3MX6 Δnup60*::*kanMX* | “” |
| MRG5788 | BY4742 *Δts(cga)c*::bleo^r^ *HOp*::*NLS-mCherry*::*URA3* *his3Δ1*::*MET25p-MS2-CP-ΔNLS-1×yeGFP*::*HIS3* pRJG1[YCplac111-*Sb-tS(CGA)C-2×MS2-hp*::*LEU2*] | “” |
| MRG5787 | MRG5788 *Δnup60*::*kanMX* | “” |
| MRG5789 | MRG5788 *Δlos1*::*hphMX* | “” |
| MRG5816 | MRG5788 *Δmlp1*::*kanMX* | “” |
| MRG5817 | MRG5788 *Δmlp1*::*TRP1 Δmlp2*::*kanMX* | “” |
| MRG7400 | MRG5788 with pRJG1 replaced by pJW031-LYS2[*tS(CGA)C/LYS2/CEN*] | “” |
| MRG7425 | *MAT***α** *RS*::*HMRE-a2a1-HMRI-GIT1*::*RS* *(LEU2*::*GAL1p-R)2*::*leu2-3,112 ADE2*::*HIS3p-GFPlacI*::*ade2-1* *Δlys2* *NIC96-mRFP*::*natMX Δnup133*::*hphMX tS(CGA)C-BUD31*::*256lac^op^-TRP1 Δmlp1*::*bleoMX Δmlp2*::*SpHIS5MX MET3p-CDC20-kanMX cir0 [pMC3 (nup133-ΔN)]* | “” |
| MRG7426 | *MAT***α** *RS*::*HMRE-a2a1-HMRI-GIT1*::*RS* *(LEU2*::*GAL1p-R)2*::*leu2-3,112 ADE2*::*HIS3p-GFPlacI*::*ade2-1* *Δlys2* *NIC96-mRFP*::*natMX Δnup133*::*hphMX tT(UGU)G1*::*256lac^op^-TRP1 Δmlp1*::*bleoMX Δmlp2*::*SpHIS5MX MET3p-CDC20-kanMX cir0 [pMC3 (nup133-ΔN)]* | “” |

**Table B** – Oligonucleotides

| Site | Name | Sequence | | Species | Use | Source |
| --- | --- | --- | --- | --- | --- | --- |
| *GIT1p* | (V) 5'-GIT1_for | 5’-GAGTG TCCGC ATGAT TAATA CTTTT CG-3’ | | *S. cerevisiae* | ChIP-qPCR | [3] |
|  | (V) 5'-GIT1_rev | 5’-ATATG AAGAT AAATG TGGCA CCAAA CG-3’ | |  |  |  |
| *tT(AGU)C* | (II) tRNA_for | 5’-CACCA ATTCC GCATC TGCAG ATTAC-3’ | | “” | “” | “” |
|  | (II) tRNA_rev | 5’-GGGTG TCACC GAATA ACGTG AT-3’ | |  |  |  |
| *tS(CGA)C* | tS(CGA)C-for | 5’-TCTAC TGTCT TGGAT CTGTG-3’ | | “” | “” | “” |
|  | tS(CGA)C-rev | 5’-GACTG CAGTG AATAG GATTG-3’ | |  |  |  |
| *tT(UGU)G1* | tT(UGU)G1_for | 5’-CTCAA ATCTG AGTCT CTTGG-3’ | | “” | “” | “” |
|  | tT(UGU)G1_rev | 5’-GATGG ACAGA ACTTC CGTAC-3’ | |  |  |  |
| *tL(CAA)N* | tL(CAA)_for | 5’-TAAGG CGCCT GATTC AAGAA A-3’ | | “” | “” | This study |
|  | tL(CAA)_rev | 5’-CTAAG AGATT CGAAC TCTTG C-3’ | |  |  |  |
| *tW(CCA)G2* | tW(CCA)_for | 5’-GCTTT CGACT CCAAT TAAAT C-3’ | | “” | “” | “” |
|  | tW(CCA)_rev | 5’-ACAGG AATTG AACCT GCAAC-3’ | |  |  |  |
| tRNA^Ser(CGA)^ | sup61_GG-Sc for | 5’-TAAGG CGAGA GACTC GAATG G-3’ | | “” | RT-qPCR | [3] |
|  | sup61 rev | 5’-GCAGG ATTTG AACCA GCG-3’ | |  |  |  |
| tRNA^Leu(CAA)^ | tL(CAA)_for | 5’-TAAGG CGCCT GATTC AAGAA A-3’ | | “” | “” | “” |
|  | tL(CAA)_rev | 5’-CTAAG AGATT CGAAC TCTTG C-3’ | |  |  |  |
| tRNA^Trp(CCA)^ | tW(CCA)_for | 5’-GCTTT CGACT CCAAT TAAAT C-3’ | | “” | “” | “” |
|  | tW(CCA)_rev | 5’-ACAGG AATTG AACCT GCAAC-3’ | |  |  |  |
| ACT1 intron | ACT1 for (intron) | 5’-GTACT AACAT CGATT GCTTC-3’ | | “” | “” | “” |
|  | ACT1 rev | 5’-AAGGA CAAAA CGGCT TGGAT-3’ | |  |  |  |
| ACT1 | ACT1 for | 5’-CGTTA CCCAA TTGAA CACGG T-3’ | | “” | “” | “” |
|  | ACT1 rev | 5’-AAGGA CAAAA CGGCT TGGAT-3’ | |  |  |  |
| MS2-CP-delNLS | delNLS for | 5’-TCAGC AATCG CAGCA AACTC CGGCA TCTAC GATGA GCTGT ACAC-3’ | | “” | plasmid construction | This study |
|  | delNLS rev | 5’-GGCCG TGTAC AGCTC ATCGT AGATG CCGGA GTTTG CTGCG ATTGC-3’ | |  |  |  |
| 1×yeGFP | 1×yeGFP for | 5’-cca CGGCC GCCGC TGCTG CCGCT GCAGC TGCTG-3’ | | “” | “” | “” |
|  | 1×yeGFP rev | 5’-cca CCGCG GTTAT TTGTA CAATT CATCC ATACC-3’ | |  |  |  |
| tRNA in plasmid | Upstream-tRNA(245)-SphI | 5’-ccacc GCATG CAAGC TGGAT GCAAG GATTG A-3’ | | “” | “” | “” |
|  | tRNA seq reverse | 5’-GACGG CCAGT GAATT CGAG-3’ | | “” | “” | “” |
| 2×MS2-hp in tS(CGA)C | YCPlac111_F2 | 5’-CGCAT CTGCA GATTA CTTTC C-3’ | | “” | “” | “” |
|  | 2×MS2-hp tail rev | 5’-aggat ttgaa ccagc gcggg cagag cccaa gagat agccg aacga catgg gtgat cctca tgtcc aaggc ctag-3’ | | “” | “” | “” |
| tRNA^Ser(CGA)^ | universal tRNA^Ser(CGA)^ LNA probe | 5’-ctta+A c+Ca+Ct cg+Gcc at-3’ | *Each of the locked nucleic acid (LNA) residues is shown with a capital letter and preceded by a “+” symbol. | “” | Northern hybridization | “” |
| 2×MS2bs | 2×MS2bs LNA probe | 5’-ctc+At +Gtcca +Ag+Gc+C tag-3’ |  | “” | “” | “” |
| tRNA^Ile(UAU)^ | pre-tRNA^Ile(UAU)^ | 5’-CGTTG CTTTT AAAGG CCTGT TTGAA AGGTC TTTGG CACAG AAACT TCGGA AACCG AATGT TGCTA T-3’ | | “” | tRNA FISH | [4] |
| U6 | U6 snRNA F | 5’-CTCGC TTCGG CAGCA CA-3’ | | *H. sapiens* | RT-qPCR | This study |
|  | U6 snRNA R | 5’-AACGC TTCAC GAATT TGCGT-3’ | |  |  |  |
| tRNA-Glu(CTC) | tRNA-Glu(CTC)_F | 5’-TCCCT GGTGG TCTAG TGGTT-3’ | | “” | “” | “” |
|  | tRNA-Glu(CTC)_R | 5’-CCTGA CCGGG AATCG AACC-3’ | |  |  |  |
| tRNA-Tyr(GTA) | tRNA-Tyr(GTA)_F | 5’-CCTTC GATAG CTCAG CTGGT-3’ | | “” | “” | “” |
|  | tRNA-Tyr(GTA)_R | 5’-ACCAG CGACC TAAGG ATCTA-3’ | |  |  |  |
| tRNA-His(GTG) | tRNA-His(GTG)_F | 5’-GCCGT GATCG TATAG TGGT-3’ | | “” | “” | “” |
|  | tRNA-His(GTG)_R | 5’-TGCCG TGACT CGGAT TCG-3’ | |  |  |  |
| tRNA-Leu(CAG) | tRNA-Leu(CAG)_F | 5’-AAGGC GCTGC GTTCA GGTCG-3’ | | “” | “” | “” |
|  | tRNA-Leu_R | 5’-GTGGG ATTCG AACCC ACGCC-3’ | |  |  |  |
| tRNA-Gly(CCC) | tRNA-Gly(CCC/GCC)_F | 5’-GCATT GGTGG TTCAG TGGTA G-3’ | | “” | “” | “” |
|  | tRNA-Gly(CCC)_R | 5’-GCATT GGCCG GGAAT TGAAC-3’ | |  |  |  |
| tRNA-Gly(GCC) | tRNA-Gly(CCC/GCC)_F | 5’-GCATT GGTGG TTCAG TGGTAG-3’ | | “” | “” | “” |
|  | tRNA-Gly(GCC/TCC)_R | 5’-GGCCG GGAAT CGAAC CCGGG-3’ | |  |  |  |
| U1 | U1 snRNA F | 5’-CCATG ATCAC GAAGG TGGTT T-3’ | | “” | “” | “” |
|  | U1 snRNA R | 5’-ATGCA GTCGA GTTTC CCACA T-3’ | |  |  |  |
| 5.8S rRNA | 5.8S rRNA F | 5’-GGTGG ATCAC TCGGC TCGT-3’ | | “” | “” | “” |
|  | 5.8S rRNA R | 5’-GCAAG TGCGT TCGAA GTGTC-3’ | |  |  |  |
| GAPDH | GAPDH_F | 5’-AGCCA CATCG CTCAG ACAC-3’ | | “” | “” | “” |
|  | GAPDH_R | 5’-GCCCA ATACG ACCAA ATCC-3’ | |  |  |  |
| pretRNA-Leu | pretRNA-Leu_F | 5’-GTCAG GATGG CCGAG TGGTC TAAG-3’ | | “” | “” | [5] |
|  | pretRNA-Leu_R | 5’-CCACG CCTCC ATACG GAGAA CCAGA AGACC C-3’ | |  |  |  |
| pretRNA-Arg | pretRNA-Arg_F | 5’-GGCTC TGTGG CGCAA TGGAT A-3’ | | “” | “” | “” |
|  | pretRNA-Arg_R | 5’-TTCGA ACCCA CAACC TTTGA ATTGC TC-3’ | |  |  |  |
| pretRNA-Tyr | tRNA-Tyr_F | 5’-CCTTC GATAG CTCAG CTGGT AGAGC GGAGG-3’ | | “” | “” | “” |
|  | tRNA-Tyr_R | 5’-CGGAA TTGAA CCAGC GACCT AAGGA TGTCC-3’ | |  |  |  |
| TPR | Tpr-siRNA (Tsi) | 5’-GCACA ACCAG GATAA GGTTA-3’ | | “” | Gene knockdown | [6] |
|  | Tpr-siRNA1 (Tsi-1) | 5’-GAAGA AGUGC GUAAG AAUA-3’ | | “” | “” | “” |
|  | Tpr-siRNA2 (Tsi-2) | 5’-GGCAU ACACU UACUA GAAA-3’ | | “” | “” | “” |
| NXF1 | SiTap-1 (Nsi-1) | 5’-GGAUAUCUAUCAUCAUCAA-3’ | | “” | “” | [7] |
|  | SiTap-2 (Nsi-2) | 5’-ACAUUGACGUUGUCCUGAA-3’ | | “” | “” | “” |

**Table C** – TPR mRNA level in the NCI-60 cell lines from GSE5720.

| **ID_REF** | **Cell line** | **Sample Name** | **Tissue Origin** | **201730_s_at** | average | SD |  | **201731_s_at** | average | SD |
| --- | --- | --- | --- | --- | --- | --- | --- | --- | --- | --- |
| GSM133565 | CCRF-CEM hg133a21 | lymphoblastic leukemia | Blood (6) | 202.1 | 260.7333 | 98.36966 |  | 244.3 | 365.45 | 220.7281 |
| GSM133623 | HL-60 hg133a21 | promyelocytic leukemia |  | 162.8 |  |  |  | 171.6 |  |  |
| GSM133570 | MOLT-4 hg133a21 | lymphoblastic leukemia |  | 177.9 |  |  |  | 242.8 |  |  |
| GSM133632 | RPMI-8226 hg133a21 | Myeloma |  | 271.2 |  |  |  | 431.4 |  |  |
| GSM133668 | SR hg133a21 | Lymphoma |  | 408.2 |  |  |  | 778.1 |  |  |
| GSM133641 | K-562 hg133a21 | myelogenous leukemia |  | 342.2 |  |  |  | 324.5 |  |  |
| GSM133558 | MCF7 hg133a31 | breast epithelial adenocarcinoma | Breast (5) | 141.4 | 212.22 | 49.77778 |  | 253.9 | 438.9 | 213.5223 |
| GSM133644 | MDA-MB-231/ATCC hg133a21 | breast epithelial adenocarcinoma |  | 259.8 |  |  |  | 728.8 |  |  |
| GSM133554 | HS578T hg133a21 | breast epithelial carcinosarcoma |  | 232.1 |  |  |  | 603.8 |  |  |
| GSM133601 | T47D hg133a21 | breast epithelial carcinoma |  | 180.5 |  |  |  | 320 |  |  |
| GSM133672 | BT-549 hg133a21 | papillary infiltrating ductal carcinoma |  | 247.3 |  |  |  | 288 |  |  |
| GSM133671 | SF-268 hg133a21 | glioblastoma undifferentiated | Central Nervous System (6) | 498.3 | 432.0167 | 53.98016 |  | 551.4 | 477.05 | 82.6673 |
| GSM133639 | SF-295 hg133a21 | glioblastoma undifferentiated |  | 466.3 |  |  |  | 490.1 |  |  |
| GSM133579 | SF-539 hg133a21 | glial neoplasm |  | 434.6 |  |  |  | 512.7 |  |  |
| GSM133597 | SNB-19 hg133a21 | glioblastoma undifferentiated |  | 390.3 |  |  |  | 363.2 |  |  |
| GSM133564 | SNB-75 hg133a21 | Astrocytoma |  | 349.5 |  |  |  | 388.2 |  |  |
| GSM133654 | U251 hg133a21 | glioblastoma undifferentiated |  | 453.1 |  |  |  | 556.7 |  |  |
| GSM133600 | COLO205 hg133a21 | adenocarcinoma | Colon (7) | 393.1 | 241.2857 | 128.0498 |  | 408 | 321.9429 | 119.8541 |
| GSM133637 | HCC-2998 hg133a21 | Carcinoma |  | 75.7 |  |  |  | 236.4 |  |  |
| GSM133592 | HCT-116 hg133a21 | carcinoma very poorly differentiated |  | 327.2 |  |  |  | 446.5 |  |  |
| GSM133653 | HCT-15 hg133a21 | adenocarcinoma |  | 261.4 |  |  |  | 350.7 |  |  |
| GSM133562 | HT29 hg133a21 | adenocarcinoma |  | 354.8 |  |  |  | 417.7 |  |  |
| GSM133663 | KM12 hg133a21 | adenocarcinoma |  | 83.3 |  |  |  | 111.1 |  |  |
| GSM133621 | SW-620 hg133a21 | carcinoma |  | 193.5 |  |  |  | 283.2 |  |  |
| GSM133577 | 786-0 hg133a21 | adenocarcinoma | Kidney (8) | 151.9 | 251.6 | 90.41466 |  | 149 | 234.3125 | 85.26318 |
| GSM133626 | A498 hg133a21 | adenocarcinoma |  | 216.4 |  |  |  | 150.8 |  |  |
| GSM133550 | ACHN hg133a21 | renal cell carcinoma |  | 286 |  |  |  | 245.1 |  |  |
| GSM133657 | CAKI-1 hg133a21 | clear cell carcinoma |  | 129.9 |  |  |  | 162.5 |  |  |
| GSM133584 | RXF-393 hg133a21 | hypernephroma |  | 374.4 |  |  |  | 334.3 |  |  |
| GSM133594 | SN12C hg133a21 | renal cell carcinoma |  | 338.7 |  |  |  | 336.9 |  |  |
| GSM133561 | TK-10 hg133a21 | renal spindle cell carcinoma |  | 197.3 |  |  |  | 175.6 |  |  |
| GSM133638 | UO-31 hg133a21 | renal cell carcinoma |  | 318.2 |  |  |  | 320.3 |  |  |
| GSM133568 | A549/ATCC hg133a21 | adenocarcinoma-poor or moderately differentiated | Lung (8) | 274.1 | 344.3375 | 91.87639 |  | 253 | 338.0625 | 130.9443 |
| GSM133581 | EKVX hg133a21 | adenocarcinoma-moderately differentiated |  | 297.9 |  |  |  | 306.6 |  |  |
| GSM133591 | HOP-62 hg133a21 | adenocarcinoma-undifferentiated |  | 319 |  |  |  | 204.2 |  |  |
| GSM133602 | HOP-92 hg133a21 | large cell-undifferentiated |  | 272.4 |  |  |  | 192.3 |  |  |
| GSM133642 | NCI-H226 hg133a21 | squamous cell carcinoma |  | 413.1 |  |  |  | 445.9 |  |  |
| GSM133576 | NCI-H322M hg133a21 | small cell bronchioalveolar carcinoma |  | 476.1 |  |  |  | 420.7 |  |  |
| GSM133557 | NCI-H460 hg133a21 | large cell carcinoma-undifferentiated |  | 241.5 |  |  |  | 312.4 |  |  |
| GSM133625 | NCI-H522 hg133a21 | adenocarcinoma |  | 460.6 |  |  |  | 569.4 |  |  |
| GSM133567 | IGROV1 hg133a21 | cystoadenocarcinoma | Ovary (8) | 272.4 | 216.2625 | 104.7614 |  | 367.2 | 255.25 | 115.1539 |
| GSM133572 | IGROV1 hg133a31 | cystoadenocarcinoma |  | 121.2 |  |  |  | 124.2 |  |  |
| GSM133559 | OVCAR-3 hg133a21 | adenocarcinoma |  | 258.3 |  |  |  | 267.4 |  |  |
| GSM133616 | OVCAR-4 hg133a21 | adenocarcinoma |  | 287.9 |  |  |  | 346.1 |  |  |
| GSM133614 | OVCAR-5 hg133a21 | adenocarcinoma |  | 351.4 |  |  |  | 369 |  |  |
| GSM133655 | OVCAR-8 hg133a21 | carcinoma |  | 272.3 |  |  |  | 321.1 |  |  |
| GSM133609 | SK-OV-3 hg133a21 | adenocarcinoma |  | 61.2 |  |  |  | 86.6 |  |  |
| GSM133658 | NCI-ADR-RES hg133a21 | adenocarcinoma |  | 105.4 |  |  |  | 160.4 |  |  |
| GSM133661 | PC-3 hg133a21 | prostate adenocarcinoma | Prostate (2) | 103.4 | 171.7 | 96.59079 |  | 87.2 | 182.4 | 134.6331 |
| GSM133598 | DU-145 hg133a21 | carcinoma |  | 240 |  |  |  | 277.6 |  |  |
| GSM133662 | LOXIMVI hg133a21 | amelanotic melanoma | Skin (10) | 422.1 | 296.89 | 147.3863 |  | 465.4 | 333.78 | 161.3209 |
| GSM133634 | MALME-3M hg133a21 | malignant melanoma |  | 405.2 |  |  |  | 424.2 |  |  |
| GSM133604 | M14 hg133a21 | melanoma-melanotic |  | 136.8 |  |  |  | 124 |  |  |
| GSM133555 | SK-MEL-2 hg133a21 | melanoma |  | 186.9 |  |  |  | 181.7 |  |  |
| GSM133636 | SK-MEL-28 hg133a21 | melanoma |  | 339.1 |  |  |  | 557.2 |  |  |
| GSM133649 | SK-MEL-5 hg133a21 | melanoma |  | 277.5 |  |  |  | 392.5 |  |  |
| GSM133599 | UACC-257 hg133a21 | melanoma-melanotic |  | 594.6 |  |  |  | 546.6 |  |  |
| GSM133603 | UACC-62 hg133a21 | melanoma |  | 291.2 |  |  |  | 227.9 |  |  |
| GSM133578 | MDA-N hg133a21 | melanoma |  | 137.4 |  |  |  | 227.2 |  |  |
| GSM133628 | MDA-MB-435 hg133a21 | melanoma |  | 178.1 |  |  |  | 191.1 |  |  |

**Table D** – TPR mRNA level in the NCI-60 cell lines from GSE5846.

| **ID_REF** | **Cell line** | **Tissue Origin** | **201730_s_at** | average | SD |  | **201731_s_at** | average | SD |
| --- | --- | --- | --- | --- | --- | --- | --- | --- | --- |
| GSM136266 | CCRF-CEM | Leukemia (6) | 9.219328 | 9.041219 | 0.213134 |  | 8.940458 | 8.986548 | 0.333772 |
| GSM136267 | HL-60(TB) |  | 8.942566 |  |  |  | 8.768739 |  |  |
| GSM136268 | MOLT-4 |  | 8.808458 |  |  |  | 8.669181 |  |  |
| GSM136269 | RPMI-8226 |  | 9.081346 |  |  |  | 8.828797 |  |  |
| GSM136270 | SR |  | 8.849329 |  |  |  | 9.589267 |  |  |
| GSM136271 | K-562 |  | 9.346286 |  |  |  | 9.122848 |  |  |
| GSM136272 | MCF7 | Breast Cancer (5) | 8.259082 | 8.032551 | 0.800039 |  | 8.700114 | 8.771437 | 0.159133 |
| GSM136274 | MDA-MB-231/ATCC | | 7.324063 |  |  |  | 8.754187 |  |  |
| GSM136275 | HS 578T |  | 7.140088 |  |  |  | 8.55278 |  |  |
| GSM136277 | T-47D |  | 8.355087 |  |  |  | 8.946299 |  |  |
| GSM136279 | BT-549 |  | 9.084435 |  |  |  | 8.903804 |  |  |
| GSM136280 | SF-268 | Brain and CNS Cancer (6) | 9.430423 | 8.87963 | 0.390958 |  | 8.82088 | 8.516738 | 0.273907 |
| GSM136281 | SF-295 |  | 8.343535 |  |  |  | 8.135089 |  |  |
| GSM136282 | SF-539 |  | 8.998386 |  |  |  | 8.429037 |  |  |
| GSM136283 | SNB-19 |  | 8.636996 |  |  |  | 8.328908 |  |  |
| GSM136284 | SNB-75 |  | 9.151631 |  |  |  | 8.819109 |  |  |
| GSM136285 | U251 |  | 8.716808 |  |  |  | 8.567407 |  |  |
| GSM136286 | COLO 205 | Colorectal Cancer (7) | 8.038305 | 8.14929 | 0.916052 |  | 7.972088 | 8.290364 | 0.348636 |
| GSM136287 | HCC-2998 |  | 6.355723 |  |  |  | 7.796405 |  |  |
| GSM136288 | HCT-116 |  | 8.869583 |  |  |  | 8.825559 |  |  |
| GSM136289 | HCT-15 |  | 8.361735 |  |  |  | 8.484846 |  |  |
| GSM136290 | HT29 |  | 9.138557 |  |  |  | 8.439679 |  |  |
| GSM136291 | KM12 |  | 7.777971 |  |  |  | 8.124306 |  |  |
| GSM136292 | SW-620 |  | 8.503157 |  |  |  | 8.389663 |  |  |
| GSM136293 | 786-0 | Kidney Cancer (8) | 8.969622 | 8.903029 | 0.294 |  | 8.697258 | 8.597026 | 0.169367 |
| GSM136294 | A498 |  | 8.646313 |  |  |  | 8.3005 |  |  |
| GSM136295 | ACHN |  | 9.49399 |  |  |  | 8.763949 |  |  |
| GSM136296 | CAKI-1 |  | 8.949575 |  |  |  | 8.78581 |  |  |
| GSM136297 | RXF 393 |  | 9.075383 |  |  |  | 8.553239 |  |  |
| GSM136298 | SN12C |  | 8.811662 |  |  |  | 8.495396 |  |  |
| GSM136299 | TK-10 |  | 8.692091 |  |  |  | 8.706165 |  |  |
| GSM136300 | UO-31 |  | 8.585594 |  |  |  | 8.473894 |  |  |
| GSM136301 | A549/ATCC | Lung Cancer (9) | 8.716969 | 8.892083 | 0.335284 |  | 8.662638 | 8.662887 | 0.252799 |
| GSM136302 | EKVX |  | 8.885201 |  |  |  | 8.863557 |  |  |
| GSM136303 | HOP-62 |  | 8.983519 |  |  |  | 8.96662 |  |  |
| GSM136304 | HOP-92 |  | 8.277953 |  |  |  | 8.215805 |  |  |
| GSM136305 | NCI-H226 |  | 8.736857 |  |  |  | 8.327544 |  |  |
| GSM136306 | NCI-H23 |  | 9.312489 |  |  |  | 8.829535 |  |  |
| GSM136307 | NCI-H322M |  | 9.024774 |  |  |  | 8.737776 |  |  |
| GSM136308 | NCI-H460 |  | 8.715654 |  |  |  | 8.554079 |  |  |
| GSM136309 | NCI-H522 |  | 9.375327 |  |  |  | 8.808426 |  |  |
| GSM136310 | IGROV1 | Ovarian Cancer (7) | 8.917874 | 8.709276 | 0.430539 |  | 8.929763 | 8.535889 | 0.295117 |
| GSM136311 | OVCAR-3 |  | 8.367081 |  |  |  | 8.152924 |  |  |
| GSM136312 | OVCAR-4 |  | 9.029999 |  |  |  | 8.630773 |  |  |
| GSM136313 | OVCAR-5 |  | 9.057834 |  |  |  | 8.564847 |  |  |
| GSM136314 | OVCAR-8 |  | 9.179342 |  |  |  | 8.753646 |  |  |
| GSM136315 | SK-OV-3 |  | 8.190767 |  |  |  | 8.134496 |  |  |
| GSM136273 | NCI/ADR-RES |  | 8.222035 |  |  |  | 8.584773 |  |  |
| GSM136316 | PC-3 | Prostate Cancer (2) | 7.332648 | 7.88261 | 0.777764 |  | 7.410781 | 7.975875 | 0.799163 |
| GSM136317 | DU-145 |  | 8.432572 |  |  |  | 8.540968 |  |  |
| GSM136318 | LOX IMVI | Melanoma (10) | 8.367621 | 8.407403 | 0.40664 |  | 8.153384 | 8.308595 | 0.15192 |
| GSM136319 | MALME-3M |  | 8.892637 |  |  |  | 8.423515 |  |  |
| GSM136320 | M14 |  | 8.927669 |  |  |  | 8.495645 |  |  |
| GSM136321 | SK-MEL-2 |  | 8.081463 |  |  |  | 8.070501 |  |  |
| GSM136322 | SK-MEL-28 |  | 8.35277 |  |  |  | 8.433444 |  |  |
| GSM136323 | SK-MEL-5 |  | 8.400663 |  |  |  | 8.434251 |  |  |
| GSM136324 | UACC-257 |  | 8.77144 |  |  |  | 8.316496 |  |  |
| GSM136325 | UACC-62 |  | 8.630033 |  |  |  | 8.358478 |  |  |
| GSM136278 | MDA-N |  | 7.7526 |  |  |  | 8.303274 |  |  |
| GSM136276 | MDA-MB-435 |  | 7.897135 |  |  |  | 8.096961 |  |  |

**Table E** – TPR protein abundance in cell lines from the NCI-60 proteome resource.

| **Cell Line** | **Tissue Origin** |  | **Unique Peptides** | **Spectral Count** | **Expression** | Average | SD |
| --- | --- | --- | --- | --- | --- | --- | --- |
| CCRFCEM | Blood (7) |  | 77 | 210 | 2.88E+07 | 3.17E+07 | 8.17E+06 |
| HL60 |  |  | 23 | 124 | 1.59E+07 |  |  |
| K562 |  |  | 59 | 156 | 2.98E+07 |  |  |
| MOLT4 |  |  | 72 | 200 | 4.13E+07 |  |  |
| RPMI8226 |  |  | 101 | 215 | 3.36E+07 |  |  |
| SR |  |  | 40 | 91 | 3.73E+07 |  |  |
| SR |  |  | 54 | 211 | 3.53E+07 |  |  |
| BT549 | Breast (5) |  | 46 | 115 | 3.39E+07 | 2.86E+07 | 8.67E+06 |
| HS578T |  |  | 41 | 76 | 2.81E+07 |  |  |
| MCF7 |  |  | 77 | 256 | 4.01E+07 |  |  |
| MDAMB231 |  |  | 40 | 89 | 2.10E+07 |  |  |
| T47D |  |  | 29 | 66 | 1.96E+07 |  |  |
| SF268 | Central Nervous System (6) | | 42 | 66 | 1.16E+07 | 2.30E+07 | 9.51E+06 |
| SF295 |  |  | 65 | 233 | 2.62E+07 |  |  |
| SF539 |  |  | 69 | 202 | 3.80E+07 |  |  |
| SNB19 |  |  | 43 | 269 | 2.44E+07 |  |  |
| SNB75 |  |  | 60 | 114 | 1.39E+07 |  |  |
| U251 |  |  | 61 | 199 | 2.41E+07 |  |  |
| COLO205 | Colon (7) |  | 47 | 128 | 1.97E+07 | 2.37E+07 | 6.49E+06 |
| HCC2998 |  |  | 53 | 146 | 2.19E+07 |  |  |
| HCT116 |  |  | 61 | 189 | 2.43E+07 |  |  |
| HCT15 |  |  | 45 | 92 | 1.56E+07 |  |  |
| HT29 |  |  | 77 | 224 | 1.97E+07 |  |  |
| KM12 |  |  | 107 | 231 | 3.26E+07 |  |  |
| SW620 |  |  | 55 | 211 | 3.22E+07 |  |  |
| 786-0 | Kidney (8) |  | 42 | 130 | 2.60E+07 | 1.72E+07 | 7.75E+06 |
| A498 |  |  | 38 | 101 | 1.01E+07 |  |  |
| ACHN |  |  | 51 | 154 | 1.90E+07 |  |  |
| CAKI1 |  |  | 86 | 184 | 3.10E+07 |  |  |
| RXF393 |  |  | 53 | 126 | 1.64E+07 |  |  |
| SN12C |  |  | 39 | 87 | 9.95E+06 |  |  |
| TK10 |  |  | 58 | 123 | 1.45E+07 |  |  |
| UO31 |  |  | 59 | 123 | 1.10E+07 |  |  |
| A549 | Lung (10) |  | 41 | 107 | 1.70E+07 | 3.31E+07 | 2.12E+07 |
| EKVX |  |  | 20 | 47 | 1.14E+07 |  |  |
| H226 |  |  | 75 | 211 | 2.48E+07 |  |  |
| H23 |  |  | 58 | 205 | 3.50E+07 |  |  |
| H322M |  |  | 61 | 115 | 5.30E+07 |  |  |
| H460 |  |  | 93 | 281 | 3.93E+07 |  |  |
| H522 |  |  | 111 | 398 | 8.04E+07 |  |  |
| HOP62 |  |  | 31 | 103 | 9.49E+06 |  |  |
| HOP92 |  |  | 65 | 236 | 2.99E+07 |  |  |
| HOP92 |  |  | 58 | 196 | 3.10E+07 |  |  |
| IGROV1 | Ovary (7) |  | 90 | 228 | 3.43E+07 | 2.76E+07 | 1.19E+07 |
| OVCAR3 |  |  | 69 | 308 | 4.01E+07 |  |  |
| OVCAR4 |  |  | 73 | 311 | 4.30E+07 |  |  |
| OVCAR5 |  |  | 31 | 74 | 1.07E+07 |  |  |
| OVCAR8 |  |  | 63 | 179 | 2.14E+07 |  |  |
| SKOV3 |  |  | 52 | 101 | 1.79E+07 |  |  |
| NCI-ADR-RES |  |  | 88 | 210 | 2.58E+07 |  |  |
| DU145 | Prostate (2) |  | 62 | 155 | 1.67E+07 | 1.59E+07 | 1.06E+06 |
| PC3 |  |  | 44 | 105 | 1.52E+07 |  |  |
| LOXIMVI | Skin (9) |  | 29 | 87 | 2.87E+07 | 2.49E+07 | 1.13E+07 |
| M14 |  |  | 73 | 166 | 1.96E+07 |  |  |
| MALME3M |  |  | 59 | 180 | 2.29E+07 |  |  |
| SKMEL2 |  |  | 77 | 400 | 4.37E+07 |  |  |
| SKMEL28 |  |  | 37 | 116 | 1.36E+07 |  |  |
| SKMEL5 |  |  | 71 | 194 | 2.12E+07 |  |  |
| UACC257 |  |  | 70 | 271 | 3.82E+07 |  |  |
| UACC62 |  |  | 67 | 229 | 2.86E+07 |  |  |
| MDAMB435 |  |  | 13 | 27 | 7.67E+06 |  |  |

**Table F** – Correlation analysis of TPR mRNA level and clinicopathological features in lung cancer patients.

| Variable | n | TPR mRNA level | | χ^2^ | *P* |
| --- | --- | --- | --- | --- | --- |
|  |  | High (%) | Low (%) |  |  |
| Age |  |  |  |  |  |
| <70 | 210 | 107 (51.0) | 103 (49.0) | 2.10×10^-4^ | 0.988 |
| ≥70 | 96 | 49 (51.0) | 47 (49.0) |  |  |
| Gender |  |  |  |  |  |
| Male | 186 | 98 (52.7) | 88 (47.3) | 1.76 | 0.185 |
| Female | 82 | 36 (43.9) | 46 (56.1) |  |  |
| Caner type |  |  |  |  |  |
| Adenocarcinoma | 131 | 60 (45.8) | 71 (54.2) | 1.31 | 0.253 |
| Squamous cell carcinoma | 205 | 107 (52.2) | 98 (47.8) |  |  |
| Smoking |  |  |  |  |  |
| Yes | 220 | 116 (52.7) | 104 (47.3) | 4.08 | **0.0433^*^** |
| Never | 42 | 15 (35.7) | 27 (64.3) |  |  |
| Grade |  |  |  |  |  |
| I | 36 | 11 (30.6) | 25 (69.4) | 8.26 | **0.0161^*^** |
| II | 191 | 100 (52.4) | 91 (47.6) |  |  |
| III | 65 | 39 (60.0) | 26 (40.0) |  |  |
| Stage |  |  |  |  |  |
| I | 180 | 84 (46.7) | 96 (53.3) | 4.76 | 0.190 |
| II | 66 | 40 (60.6) | 26 (39.4) |  |  |
| III | 54 | 28 (51.9) | 26 (48.1) |  |  |
| IV | 1 | 1 (100) | 0 (0.00) |  |  |
| Size of the primary tumor |  |  |  |  |  |
| T1/T2 (≤50 mm) | 260 | 128 (49.2) | 132 (50.8) | 2.03 | 0.154 |
| T3/T4 (>50 mm) | 48 | 29 (60.4) | 19 (39.6) |  |  |
| Regional lymph node metastasis |  |  |  |  |  |
| N0 | 209 | 99 (47.4) | 110 (52.6) | 3.16 | 0.0753 |
| N1/N2/N3 | 96 | 56 (58.3) | 40 (41.7) |  |  |

**Supplementary References**

1. Thomas B, Rothstein R. Elevated recombination rates in transcriptionally active DNA. Cell. 1989;56(4):619-30.

2. Baker Brachmann C, Davies A, Cost GJ, Caputo E, Li J, Hieter P, et al. Designer deletion strains derived from Saccharomyces cerevisiae S288C: A useful set of strains and plasmids for PCR-mediated gene disruption and other applications. Yeast. 1998;14(2):115-32. doi: 10.1002/(sici)1097-0061(19980130)14:2<115::Aid-yea204>3.0.Co;2-2.

3. Chen M, Gartenberg MR. Coordination of tRNA transcription with export at nuclear pore complexes in budding yeast. Genes & Dev. 2014;28(9):959-70. doi: 10.1101/gad.236729.113.

4. Sarkar S, Hopper AK. tRNA nuclear export in saccharomyces cerevisiae: in situ hybridization analysis. Mol Biol Cell. 1998;9(11):3041-55. Epub 1998/11/05. doi: 10.1091/mbc.9.11.3041. PMID: 9802895; PMCID: PMC25586.

5. Khattar E, Kumar P, Liu CY, Akıncılar SC, Raju A, Lakshmanan M, et al. Telomerase reverse transcriptase promotes cancer cell proliferation by augmenting tRNA expression. The Journal of Clinical Investigation. 2016;126(10):4045-60. doi: 10.1172/jci86042.

6. Rajanala K, Nandicoori VK. Localization of Nucleoporin Tpr to the Nuclear Pore Complex Is Essential for Tpr Mediated Regulation of the Export of Unspliced RNA. PLoS One. 2012;7(1):e29921. doi: 10.1371/journal.pone.0029921.

7. Katahira J, Inoue H, Hurt E, Yoneda Y. Adaptor Aly and co-adaptor Thoc5 function in the Tap-p15-mediated nuclear export of HSP70 mRNA. The EMBO Journal. 2009;28(5):556-67. doi: 10.1038/emboj.2009.5.
